# A Bimetal Fe/Mg Immobilized on N‑Doped Biochar for Efficient Adsorption of Paracetamol: Performance Assessment, Mechanistic Exploration, and Density Functional Theory

**DOI:** 10.1021/acsomega.6c01218

**Published:** 2026-06-26

**Authors:** Mohammed A. Al-Haiqi, Choon-Fu Goh, Wen-Da Oh

**Affiliations:** † School of Chemical Sciences, Universiti Sains Malaysia, Gelugor, Penang 11800, Malaysia; ‡ Chemistry Department, College of Science, Hadhramout University, Mukalla 50511, Yemen; § School of Pharmaceutical Sciences, Universiti Sains Malaysia, Gelugor, Penang 11800, Malaysia

## Abstract

In this study, a series of bimetallic Fe/Mg-incorporated, N-doped biochars (Fe–Mg@N-BC) was prepared via pyrolysis of coconut shells for paracetamol (PCM) adsorption. In comparison to single-metal and N-doped biochars, Fe–Mg@N-BC exhibited significantly greater adsorption capacity, illustrating the synergistic effect of bimetallic codoping. The Fe–Mg@N-BC with an Fe content of 13.8 wt % (denoted as Fe–Mg@N-BC-3) exhibited the highest adsorption capacity, reaching 157.9 mg/g at 25 °C at 0.3 g/L adsorbent loading. The characteristics of Fe–Mg@N-BC-3 were evaluated using FTIR, XRD, XPS, SEM, and EDX, which indicated that metal atoms were uniformly distributed within the carbon layer, resulting in the formation of abundant active sites for PCM adsorption. The excellent performance of Fe–Mg@N-BC-3 was mainly attributed to the formation of favorable active sites and a larger specific surface area of 604.5 m^2^/g. The adsorption kinetics and isotherms were best described by the pseudo-second-order model (R^2^ = 0.9987) and Langmuir model (R^2^=0.9998), respectively. The effects of Fe content, pH, adsorbent dosages, and water matrix on PCM adsorption were also investigated. Further characterization studies revealed that PCM adsorption on Fe–Mg@N-BC-3 is attributed to a combination of metal complexation, cation-π interactions, hydrogen bonding, and π-π stacking. Density functional theory calculations demonstrated that Fe–Mg@N-BC exhibited increased adsorption energy, thereby strengthening electronic interactions between the adsorbent’s surface and PCM. Overall, the high removal efficiency and structural stability of Fe–Mg@N-BC-3 highlight its potential as an effective and environmentally sustainable adsorbent for antibiotic removal from water.

## Graphical abstract

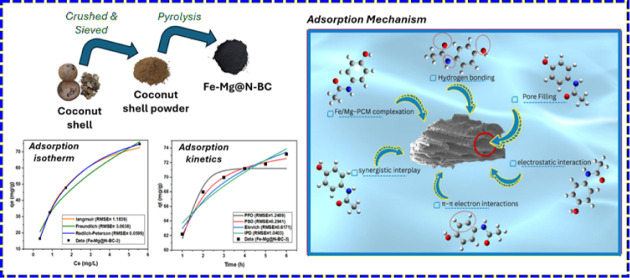

## 1 Introduction

Pharmaceutical products are among the most prevalent contaminants detected in wastewater, lakes, and rivers, largely due to their extensive consumption and inefficient removal during conventional wastewater treatment processes. Paracetamol (PCM, 4-hydroxyacetanolide—C_8_H_9_NO_2_), also known as acetaminophen, is a widely used analgesic and antipyretic pharmaceutical.^1^ PCM can persist for extended periods in water bodies. In Malaysia, PCM has been detected in surface waters at concentrations typically in the nanogram per liter range, with reported values of 346.3 ng/L in the Langat River^2^ and 1,450 ng/L in the Damansara River.^3^ In contrast, wastewater effluents in Johor Bahru have shown much higher levels, reaching up to 9,299 ng/L.^4^ The presence and persistence of PCM in water bodies highlight the inefficiency of conventional treatment processes and emphasize the urgent need for more effective removal strategies, as these contaminants pose potential risks to aquatic organisms and, ultimately, public health.^5^ Notably, PCM has been associated with confirmed ecological risks, including toxicity to aquatic organisms and potential long-term effects.^6^ Several methods have been employed for the removal of pharmaceutical contaminants, including reverse osmosis, ion exchange, ultrafiltration, ozonation, advanced oxidation processes, sedimentation, catalytic degradation, and solvent extraction. However, these approaches are often associated with high operational costs, complex operation, and the potential formation of hazardous byproducts.^7^ In contrast, adsorption has emerged as a promising alternative because of its operational simplicity, high removal efficiency, and cost-effectiveness.^8^

Sustainable carbon materials have attracted extensive multidisciplinary interest due to their distinctive physicochemical and textural properties.^9^ Specifically, porous carbon materials such as biochar (BC) are known for their high specific surface area, enhanced mass transfer, and tunable surface chemistry, which make them highly effective for environmental applications.^10^ Previous studies have demonstrated that BC is effective in removing pharmaceutical contaminants, including PCM, from aqueous environments.^11,12^ It can be produced from a diverse range of biomass sources, with coconut shells (CS) being among the most commonly utilized and effective feedstocks. CS serve as an effective precursor to prepare adsorbents for the sequestration and removal of various environmental contaminants, notably heavy metals, industrial dye effluents, pharmaceuticals, and organic pollutants, owing to their wide availability, renewability, and high adsorption capacity.^13^ CS can undergo various thermochemical processes at high temperature >500 °C under oxygen-limiting conditions to obtain BC.^14^ BC is a highly porous carbonaceous material obtained via pyrolysis and has garnered significant scientific interest due to its relatively large surface area, cost-effectiveness, environmental friendliness, and broad applicability in contaminant removal from water, thereby reducing the risk of pollution-related health issues.^15^

Despite the extensive use of BC in environmental remediation, its performance in removing pharmaceutical pollutants such as PCM remains limited due to insufficient active sites and low surface polarity. Recent studies have demonstrated that heteroatom doping, particularly N doping, is an efficient modification strategy to enhance the catalytic performance of BC.^16^ As such, the incorporation of Fe into BC derived from CS significantly enhances its structural and surface properties. Specifically, Fe modification improves porosity and enriches oxygen-containing functional groups, thereby facilitating the efficient adsorption of contaminants such as antibiotics.^17^ In addition, Fe introduces redox-active sites (Fe^2+^/Fe^3+^), which promote electron transfer and contribute to improved adsorption performance. Furthermore, Fe incorporation can increase the specific surface area and pore development of the carbon matrix, both of which are critical factors for achieving high adsorption efficiency.^18^ Meanwhile, the incorporation of metals such as Fe and Mg can enhance the redox activity and complexation capacity of BC.^19^ In a recent study, Mainali^20^ et al. revealed that Mg and N-codoped BC can be effectively used for phosphate removal from wastewater due to the greater adsorbent stability and the creation of more active sites. The presence of bimetal on the BC surface may also induce catalytic properties.^21^ Current research has largely focused on single-metal-doped BC systems, whereas studies investigating the combined effects of bimetallic incorporation and N doping on BC for PCM adsorption remain scarce. Moreover, the underlying mechanisms governing the enhanced adsorption performance such as coordination bonding, charge redistribution, and π-π interactions are not yet fully understood. Accordingly, the synergistic combination of Fe and Mg with N doping is expected to enhance the adsorption performance of PCM by providing additional active sites and facilitating interactions such as coordination bonds and π-π interactions. Therefore, a significant research gap exists in the rational design and mechanistic understanding of bimetallic-incorporated and N-doped BC for efficient PCM removal, particularly with respect to structure–function relationships.

In light of the above observations, the objectives of this study are to (i) synthesize and characterize coconut-shell-derived BC modified with bimetallic Fe and Mg together with N doping using a facile and low-cost coprecipitation method, (ii) evaluate the adsorption performance of Fe–Mg@N-BC for PCM removal from aqueous solutions under varying operational conditions and model the adsorption behavior using kinetic, isotherm, and thermodynamic approaches, and (iii) assess the stability and practical applicability of Fe–Mg@N-BC in real water matrices and elucidate the adsorption mechanisms through experimental observations and density functional theory (DFT) calculations. This study aims to establish structure–function relationships that provide mechanistic insights into the rational design of engineered BC-based adsorbents for pharmaceutical contaminant removal.

## 2 Materials and Methods

### 2.1 Chemicals

All materials used in this study are listed in the Supporting Information (SI) (Text S1).

### 2.2 Preparation of Adsorbent

Prior to use, CS was washed several times with distilled water, dried, crushed, and sieved to an average particle size of 100 mesh. Then, 10 g of processed CS was placed in an enclosed crucible and calcined in a muffle furnace at a ramping rate of 5 °C/min until 700 °C and maintained for 2 h. The enclosed crucible provides an oxygen-deficient environment for the formation of BC. Thereafter, the resultant product was collected and ground to a fine powder. A summary of the schematic illustration of the BC preparation process is presented in Figure 1.

**Figure 1:**
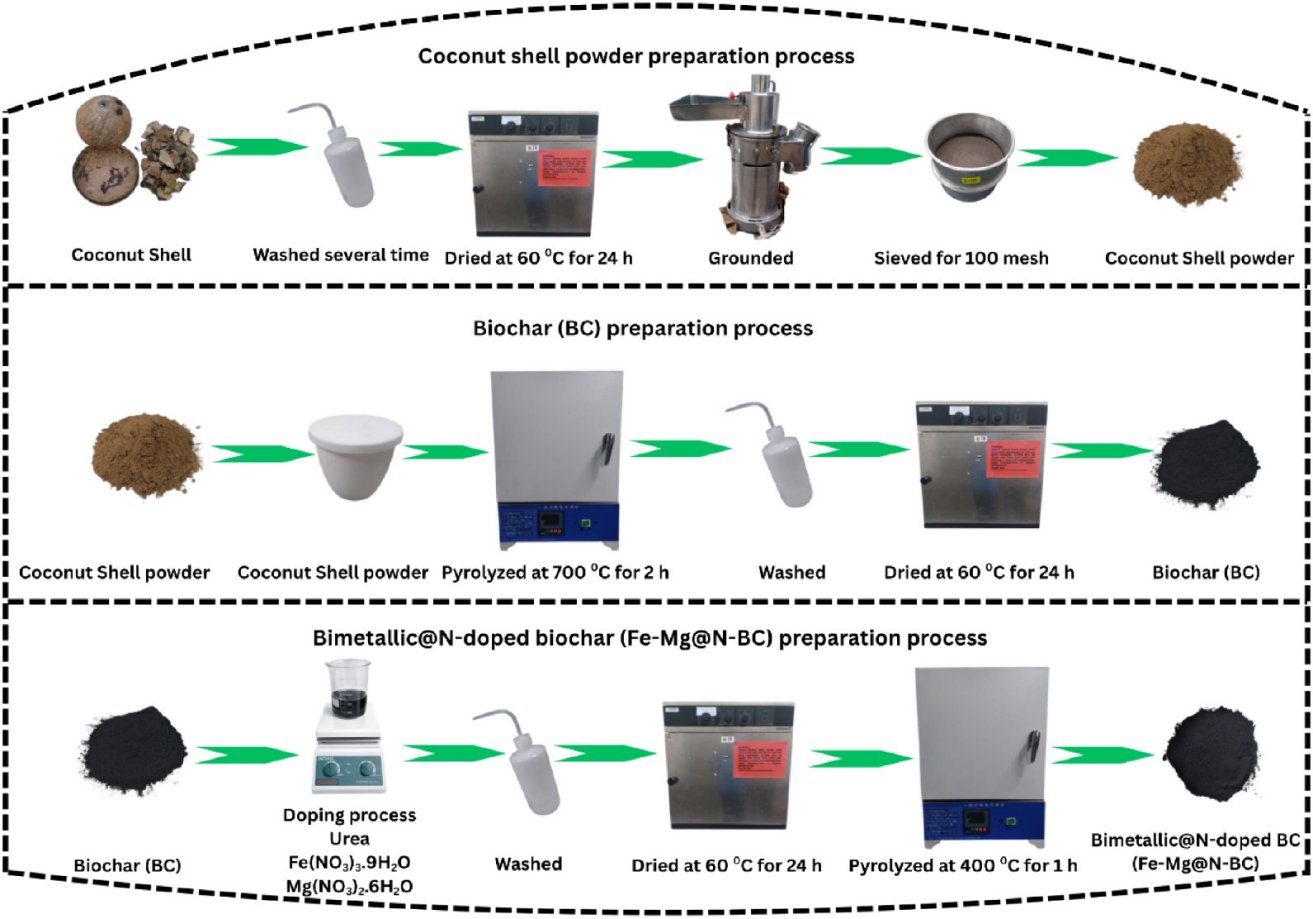
Schematic illustration of the preparation of CS powder, BC, and Fe–Mg@N-BC.

### 2.3 Bimetal-Incorporated N-Doped BC

A bimetallic-incorporated and N-codoped BC was prepared following a previously reported method,^22^ with modifications. Briefly, BC (2 g), urea (1.5 g), and metal salts corresponding to a total metal loading of 3 wt % relative to the BC mass (metal = Al, Ca, Fe, and/or Mg; aluminum nitrate, calcium nitrate, magnesium nitrate, and/or iron nitrate, respectively) were mixed into a 500 mL of distilled water and homogenized by magnetic stirring for 2 h. The resulting suspension was heated for 20 min at 100 °C, after which 30 mL of HCl was added. The mixture was then completely dried in an oven at 70–80 °C. The elevated temperatures facilitate metal incorporation into the BC matrix.^23^ Finally, the dried material was calcined at 400 °C for 1 h in a furnace. The resultant products were collected, washed several times with distilled water, and stored in a container prior to use. Depending on the type of metal present, the adsorbents were denoted as Al–Mg@N-BC, Fe–Al@N-BC, Ca–Mg@N-BC, Fe–Ca@N-BC, Ca–Al@N-BC, and Fe–Mg@N-BC-1. To investigate the influence of Fe loading, the Fe content was changed in a systematic manner, from 3 to 6.8 and 13.8 wt %. The resulting adsorbents were labeled Fe–Mg@N-BC-1, Fe–Mg@N-BC-2, and Fe–Mg@N-BC-3, respectively.

### 2.4 Characterization of Fe–Mg@N-BC-3

X-ray Diffractometer Bruker D8 Advance was used to identify mineral content and crystallinity in adsorbents using a Cu–Kα target at 40 kV and 40 mA. Fourier Transform Infrared Spectroscopy (Model: IRSpirit-T, Shimadzu) was used to evaluate the surface functional groups on the adsorbents. The surface morphology, microstructure, and elemental distribution were analyzed using ultrahigh-resolution scanning electron microscopy (UHR-SEM, Hitachi Regulus SU 8220) coupled with energy-dispersive X-ray spectroscopy (EDX, Oxford Instruments). The textural properties, including specific surface area and porosity, were determined from N_2_ adsorption–desorption isotherms using a Micromeritics ASAP 2020 porosimeter (Micromeritics, USA). X-ray photoelectron spectroscopy (XPS) was used to investigate the surface chemical composition and the oxidation states of the constituent elements using an AXIS ULTRA DLD instrument (KRATOS Analytical, UK). The pH drift method was employed to estimate the pH at the point of zero charge (pH_pzc_),^24^ in order to characterize the surface properties of the adsorbents. Briefly, 0.1 g of the adsorbent was mixed with 10 mL of 0.01 M sodium chloride (NaCl) solution, with the initial pH adjusted between 3 and 11. The suspensions were equilibrated on a mechanical shaker for 24 h. The initial pH of each mixture was precisely adjusted using 0.1 M HCl or NaOH. After equilibration, the supernatant was filtered, and the final pH was measured using a pH meter. The pH_pzc_ was determined from the plot of final pH versus initial pH by identifying the intersection point.

### 2.5 Batch Adsorption Experiments

To investigate the PCM adsorption performance, batch adsorption experiments were carried out. A stock solution with the PCM concentration of 100 mg/L was prepared by dissolving an appropriate mass of PCM in 1 L of ultrapure water. In a typical experiment, 25 mL of the desired concentration of PCM was prepared by diluting the stock solution appropriately using a flask. Then, a predetermined mass of the adsorbent was introduced into the flask, which was then agitated using an orbital shaker at 200 rpm and 25 °C. At specific time intervals, the concentration of PCM in the flask was determined, and the adsorption capacity (q_t_) and PCM removal efficiency (RE) were calculated according to eqs 1 and 2, respectively.

1 $$q_{t}=\frac{C_{0}-C_{t}}{W}V$$

2 $$\mathrm{RE\%}=\frac{C_{0}-C_{t}}{C_{0}}100$$

where C_0_ and C_t_ are the concentrations of PCM at time 0 and t, respectively, W is the mass of adsorbent (g/L), and V is the volume used (L). The concentration of PCM was quantified using a calibration curve obtained with a UV–vis absorption spectrophotometer at λ_max_ = 243 nm. Specifically, to determine the contact time, a series of flasks containing 50 mg/L of PCM and 0.6 g/L of adsorbent loading were used. At selected time intervals, i.e., 1, 2, 3, 4, 5, 6, 8, 10, 12, and 24 h, a flask was removed from the shaker, and the concentration of PCM was analyzed. Once the contact time of each adsorbent was determined, the best adsorbent was selected, and the effects of adsorbent loading (0.3, 0.6, 0.9, 1.2 g/L), pH (3, 5, 7, 9, 11), initial PCM concentration (10, 20, 30, and 50 mg/L), and temperature (25.0, 35.0, and 45.0 °C) on PCM adsorption were assessed. Kinetic studies were modeled using the pseudo-first-order (PFO), pseudo-second-order (PSO), Elovich, and intraparticle diffusion (IPD) equations. The adsorption isotherms were interpreted by applying the Langmuir, Freundlich, and Redlich–Peterson. Finally, the thermodynamic parameters, including the Gibbs free energy change (ΔG°), enthalpy change (ΔH°), and entropy change (ΔS°), were determined to elucidate the spontaneity and heat changes associated with the process. The details are provided in Table S1.

### 2.6 Water Matrix Effect and Real Water

The applicability of the adsorbent was further evaluated in the presence of competing ions under environmentally relevant conditions.^25^ Batch experiments consisting of 0.6 g/L of adsorbent in 25 mL of 50 mg/L PCM solution were conducted in the presence of the water matrix species. Individual cations (Ca^2+^, Na^+^, Mg^2+^, Fe^3+^), anions (Cl^–^, CO_3_^2–^, HCO_3_^–^, SO_4_^2–^), and their mixtures were tested at the concentrations of 0.1, 1.0, and 10 mM. Ionic strength was also studied with KCl solutions ranging between 10 and 50 mM. Meanwhile, tap, river, and seawater samples spiked with PCM (50 mg/L) were prepared to investigate the performance of Fe–Mg@N-BC-3 in complex water matrices. Tap water was collected from the School of Chemical Sciences, Universiti Sains Malaysia (USM), Pulau Pinang, Malaysia. River water was collected from Tukun River, Pulau Pinang, Malaysia, while seawater was collected from Queensbay seaside, Pulau Pinang, Malaysia. The adsorption experiments were conducted with 0.6 g/L adsorbent under two pH conditions: at the ambient pH of the water samples (pH = 8.43, 6.31, and 6.54 for tap, river, and seawater, respectively) and at pH 7. After reaching equilibrium (24 h), samples were filtered, and residual PCM concentration was measured by using UV–vis spectrophotometer.

### 2.7 Regeneration and Recyclability Studies

The economic viability of an adsorbent is, essentially, related to its potential for regeneration and reutilization. In the initial screening, 0.1 N HCl, 20% (v/v) ethanol, and 20% (v/v) methanol were identified as the most effective desorption agents. Accordingly, the experimental protocol was performed by first contacting Fe–Mg@N-BC-3 with PCM. This was realized by contacting 0.6 g/L of Fe–Mg@N-BC-3 with 25 mL of a 50 mg/L PCM solution at a pH of 7. It was then agitated at 200 rpm for a period of 8 h to achieve adsorption equilibrium. The spent Fe–Mg@N-BC-3 was then subjected to the desorption process under identical conditions: stirring 0.6 g/L of the spent Fe–Mg@N-BC-3 in 25 mL of a desorbing agent for 8 h at 200 rpm. Subsequently, the regenerated Fe–Mg@N-BC-3 was recovered via filtration, dried at 50 °C for 12 h, and subjected to another cycle of the adsorption process. The Fe^3+^ and Mg^2+^ ions leaching from Fe–Mg@N-BC-3 were also quantified using an atomic absorption spectroscopy (AAS, PerkinElmer AAS 200) at pH 3–7 to evaluate the stability of the adsorbent. The conditions for Fe^3+^ and Mg^2+^ ions leaching study were as follows: adsorbent loading = 2 g/L, contact time = 24 h, and agitation speed = 200 rpm. After the shaking period, the samples were filtered, and the resulting filtrate was collected for analysis.

### 2.8 Density Functional Theory (DFT) Calculations

Density functional theory (DFT) calculations for PCM and adsorbent were performed using the CASTEP code (BIOVIA Materials Studio 2023). The generalized gradient approximation (GGA) with the Perdew–Burke–Ernzerhof (PBE) functional was employed to describe the exchange of the correlation energy. Dispersion interactions were included via the DFT+D: semiempirical dispersion correction with the Grimme G06 scheme. Core–valence interactions were treated using ultrasoft OTFG pseudopotentials in reciprocal space. A plane-wave cutoff energy of 400 eV and k-point (4-way) efficiency rating: very good (82%). Geometry optimization was carried out using the LBFGS algorithm with the fixed basis quality cell method until the max ionic force tolerance for BC 0.3000 × 10^–01^ eV/atom, and total energy convergence tolerance was 0.1000 × 10^–04^ eV/atom.

## 3 Results and Discussion

### 3.1 Characterization of Fe–Mg@N-BC-3

The surface morphology of the as-synthesized BCs was characterized by SEM, and the results are presented in Figure 2a. Overall, pristine BC exhibits a less porous structure compared to N-BC (Figure 2b) and Fe–Mg@N-BC-3. It is observed that the porosity of BC increases after N doping, which can be attributed to the incorporation of N atoms into the carbon lattice. This incorporation disrupts the chemical inertness of the carbon framework and induces substantial structural defects.^26^ Meanwhile, Fe–Mg@N-BC-3 in Figure 2c reveals the effectiveness of the doping and metal incorporation in inducing a morphological transformation on the traditionally rough surface of natural BC. A more open structure appeared with deeper and more pronounced channels and cracks. This is due to the effects of chemical surface corrosion resulting from the presence of Mg^2+^ and Fe^3+^ during preparation, which created a structure with an enhanced porosity. Multielement incorporation into BC can produce a more developed porous architecture and spherical particle morphology than single-element incorporation, leading to enhancement in BC porosity and structure.^27^ This morphology of Fe–Mg@N-BC-3 significantly enhances its capacity and aids the adsorption process of PCM.

**Figure 2:**
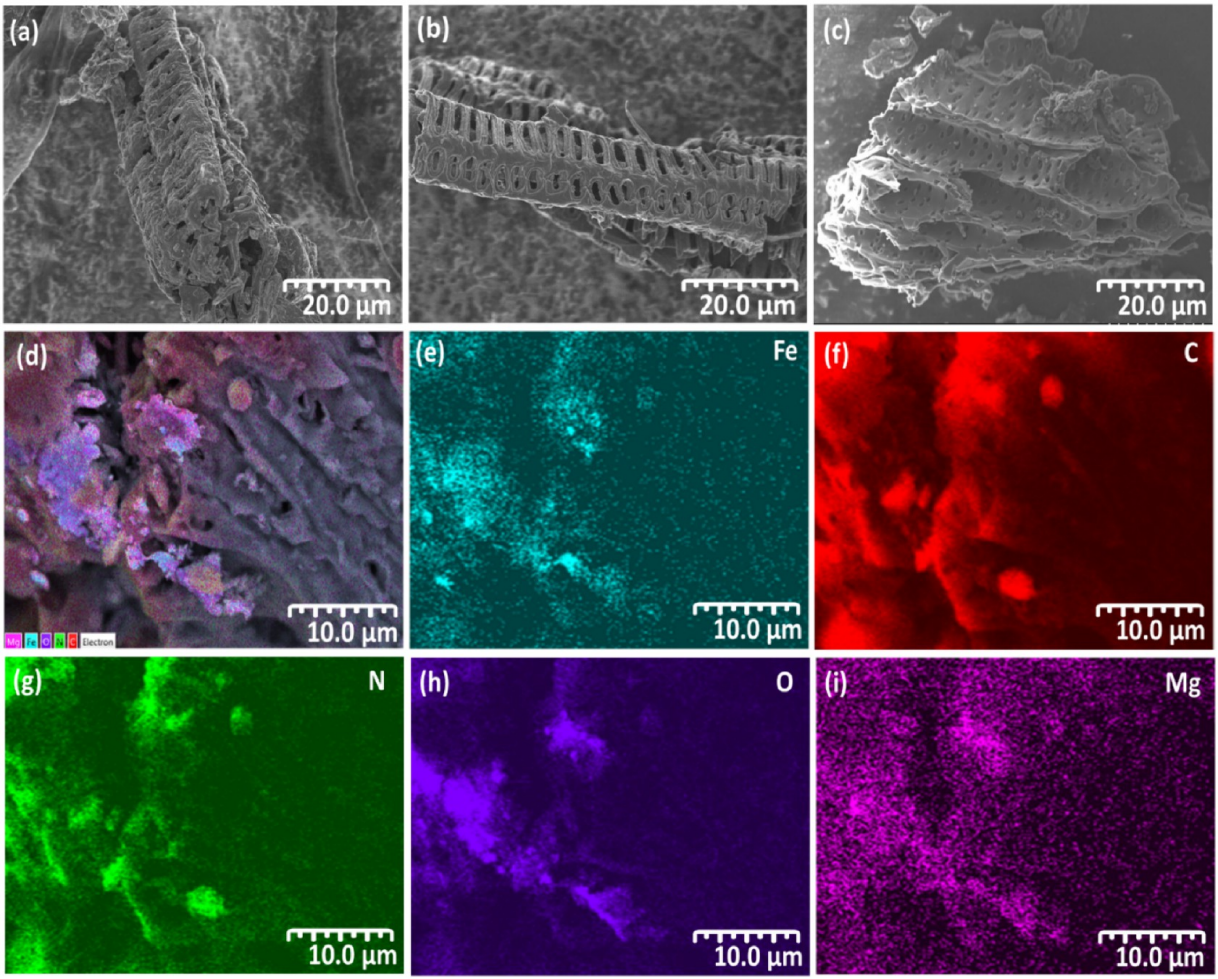
SEM of (a) BC, (b) N-BC, and (c) Fe–Mg@N-BC-3, and EDX spectrum and elemental mapping: (d) overall spectrum of Fe–Mg@N-BC-3, and (e–i) elemental mapping images of Fe, C, N, O, and Mg, respectively.

The EDX elemental mapping of Fe–Mg@N-BC-3 is presented in Figure 2d–i, revealing a well-distributed Fe, Mg, and N at 4.5, 0.7, and 2.5 wt %, respectively. This observation confirms the successful incorporation of Fe, Mg, and N onto the BC matrix with uniform dispersion, which is critical for adsorption. The textural properties of the BC, N-BC, and Fe–Mg@N-BC-3, including specific surface area and pore structure, were analyzed using N_2_ adsorption–desorption measurements. Figure 3a shows the N_2_ adsorption–desorption isotherms of BC, N-BC, and Fe–Mg@N-BC-3. As shown in Figure 3a, the isotherms for all samples were classified as Type IV with H3-type hysteresis loops, indicating the presence of a mesoporous structure, which is characteristic of BC, N-BC, and Fe–Mg@N-BC-3. The specific surface areas and total pore volumes of the doped samples were significantly enhanced compared to the BC. Notably, N-BC and Fe–Mg@N-BC-3 possessed substantially larger specific surface areas (501 m^2^/g and 604.5 m^2^/g, respectively) and pore volumes (0.254 cm^3^/g and 0.305 cm^3^/g, respectively) than BC (380.4 m^2^/g, 0.190 cm^3^/g). N doping in N-BC introduces various N groups into the carbon lattice, resulting in various nitrogenous configurations and the generation of effective structural defects.^19,28^ These defects contribute to an increased number of active sites on the surface and an increased surface area, which in turn improves the adsorption capacity of the BC. Similarly, the increased surface area of Fe–Mg@N-BC-3 is attributed to nitrogen’s ability to inhibit the agglomeration of metal particles, resulting in a more open and uniform porous structure. This effect is enhanced by the synergistic doping of nitrogen and the incorporation of bimetals (Fe/Mg) in Fe–Mg@N-BC-3, which modify the physical and chemical properties and surface functional groups, ultimately yielding an abundant pore structure, a high surface area, and abundant active sites, and facilitating PCM diffusion into the interior Fe–Mg@N-BC-3.^29^

**Figure 3:**
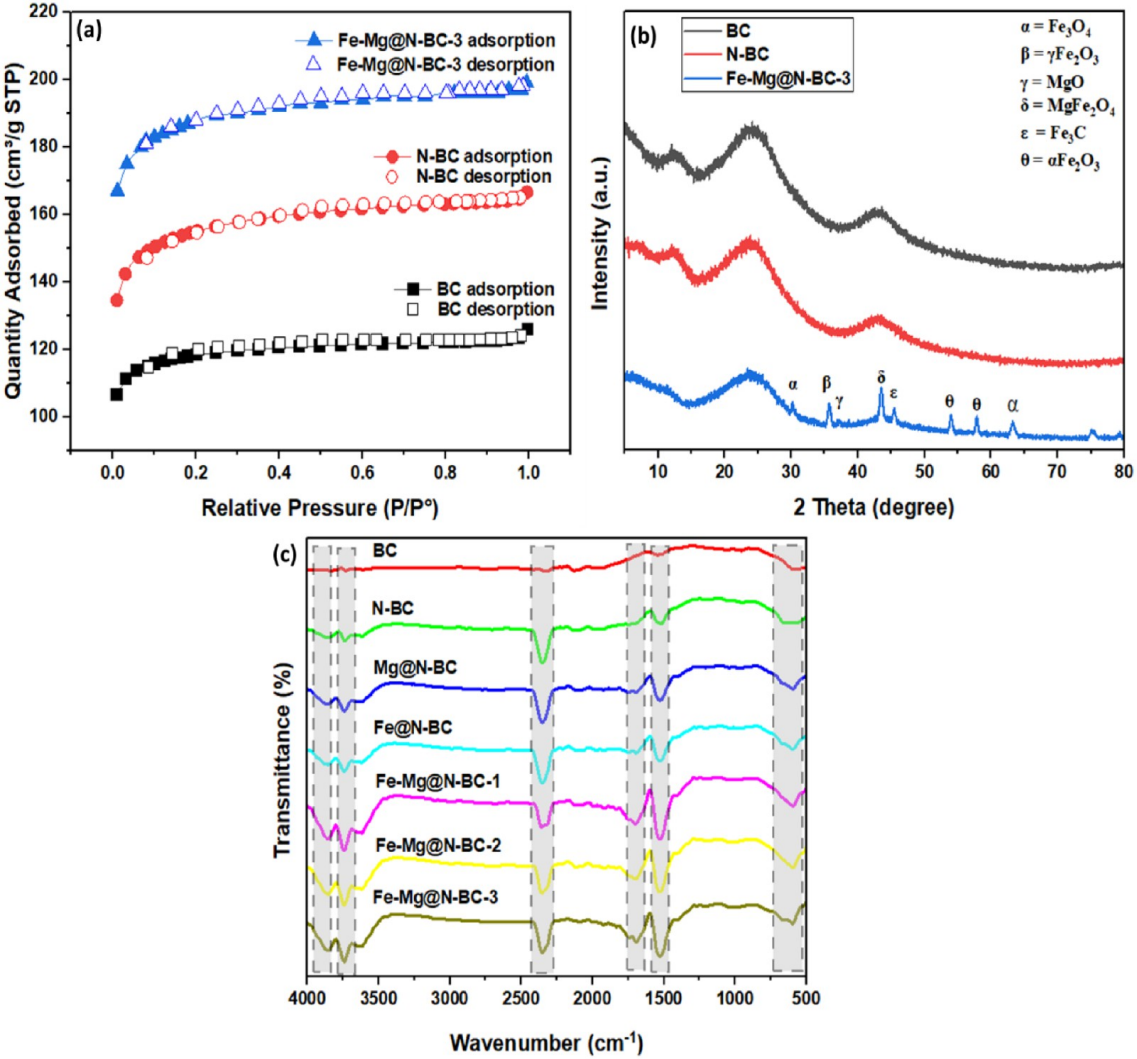
(a) N_2_ adsorption–desorption isotherms of BC, N-BC, and Fe–Mg@N-BC-3, (b) XRD patterns of BC, N-BC, and Fe–Mg@N-BC-3, and (c) FTIR spectra of adsorbents before the adsorption of PCM.

The XRD patterns of the adsorbents in Figure 3b reveal broad peaks at 2θ values of ∼24° and ∼42°, which is associated with the signature of amorphous carbon/partial graphitic domains in the sample. This indicates that the carbon matrix retains a partially amorphous character with the presence of local graphitic domains.^30^ Several diffraction peaks corresponding to mineral phases or crystalline impurities were observed. The peak at 30.2° can be assigned to Fe_3_O_4_ (PDF No. 88–0315)^31^ while the peak at 35.8° corresponds to the (311) plane of Fe_3_O_4_.^32^ The diffraction peak at 37.1° is attributed to MgO,^33^ and the peak at 43.1° is assigned to the characteristic (400) plane of MgFe_2_O_4_.^34^ The peak at approximately 45.9° corresponds to Fe_3_C.^35^ In addition, peaks at 54.2° and 57.8° are consistent with the α-Fe_2_O_3_ phase,^36^ whereas the peak at 63.2° is in agreement with Fe_3_O_4_.^32^ The diffraction peak at 75.0° is attributed to the (302) crystal plane of SiO_2_ (PDF No. 070–3755).^37^ A systematic shift of the (002) diffraction peak from approximately 24° to 24.5° and 25° in samples BC, N-BC, and Fe–Mg@N-BC-3, respectively, suggests the influence of N and bimetal incorporation in influencing the BC structure.^38^ This shift is attributed to variations in the strain fields associated with lattice defects generated during the N doping process^39^ and the successful incorporation of bimetallic sites within the carbon lattice.

As shown in Figure 3c, the FTIR spectra reveal a progressive evolution of surface chemistry from BC to Mg@N-BC, Fe@N-BC, and Fe–Mg@N-BC-3 several distinct bands reflecting the diversity of surface functional groups resulting from the introduction of Me and N. All the adsorbents have broad bands between 3500 and 3740 cm^–1,^ clearly indicating the presence of O–H and N–H bonds.^40^ The peaks in the range 3820–3935 cm^–1^ may reflect the presence of additional stretching vibrations of N–H/O–H groups or super-stretching of metal-bound hydroxyls, which are signs of a surface rich in active sites. The band at 2360 cm^–1^ typically represents the adsorption of CO_2_, a common occurrence in carbon materials with Me due to interaction with air.^41^ The peak at 1510 cm^–1^ attributed to aromatic C = C vibrations^42^ with a possible contribution from C–N bonds, reflecting the presence of nitrogenous components doped within the carbon structure. This confirms that upon introducing Me and N, the emergence of C–N stretching vibrations suggests increased surface polarity, facilitating stronger hydrogen bonding with both the phenolic −OH and amide (—NH—C=O) groups of PCM, while Mg^2+^ species may further contribute through cation-bridging effects. The band at 1043 cm^–1^, indicates C–O vibrations, which are likely due to oxygen groups on the surface of BC.^42^ The bands at (400–800 cm^–1^) attributed to M-O (Fe–O, Mg–O) vibrations, confirming the presence of metal oxides bound to the carbon surface.^40,43^ Also, the clear broad peak at 590 cm^–1^ may be due to the combination of iron oxides and MgFe_2_O_4_,^42^ which indicate the formation of Me–N coordination sites. These sites promote complexation and partial electron transfer with the amide functionality of PCM, leading to enhanced adsorption. The Fe–Mg@N-BC exhibits a synergistic amplification of these spectral features, including stronger −OH/–NH vibrations, more pronounced C–N/C=N bands, and intensified metal-N-related signals, reflecting a higher density and better organization of active sites. With increasing Fe content (Fe–Mg@N-BC-1, Fe–Mg@N-BC-2, and Fe–Mg@N-BC-3), the dominance of metal-coordinated N functionalities indicates that PCM adsorption becomes increasingly governed by a cooperative mechanism involving hydrogen bonding, cation bridging, and metal-assisted surface complexation. This synergistic interaction framework rationalizes the superior adsorption performance of the Fe–Mg@N-BC-3 sample and confirms that the enhanced removal efficiency originates from the combined chemical functionality rather than from physical adsorption alone.

### 3.2 Adsorption Performance

#### 3.2.1 Effects of Me and N on the Adsorption Performance

As shown in Figure 4a, BC exhibited the lowest adsorption capacity at 56.7 mg/g, which can be attributed to a less porous structure as shown in the SEM results. In previous studies, unmodified BC has shown varying adsorption efficiencies for PCM, ranging from 7.3 mg/g^44^ to 45.45 mg/g.^12^ Among all samples, the best performance was recorded for Fe–Mg@N-BC-3. This superior performance can be attributed to a synergistic interaction between Mg^2+^ and Fe^3+^ species,^45^ as well as to high porosity and a more open structure, the largest number of active sites, and a significant increase in surface charge and electrostatic interactions, as shown in the characterization, resulting in the highest removal efficiency of PCM from the aqueous solution.

**Figure 4:**
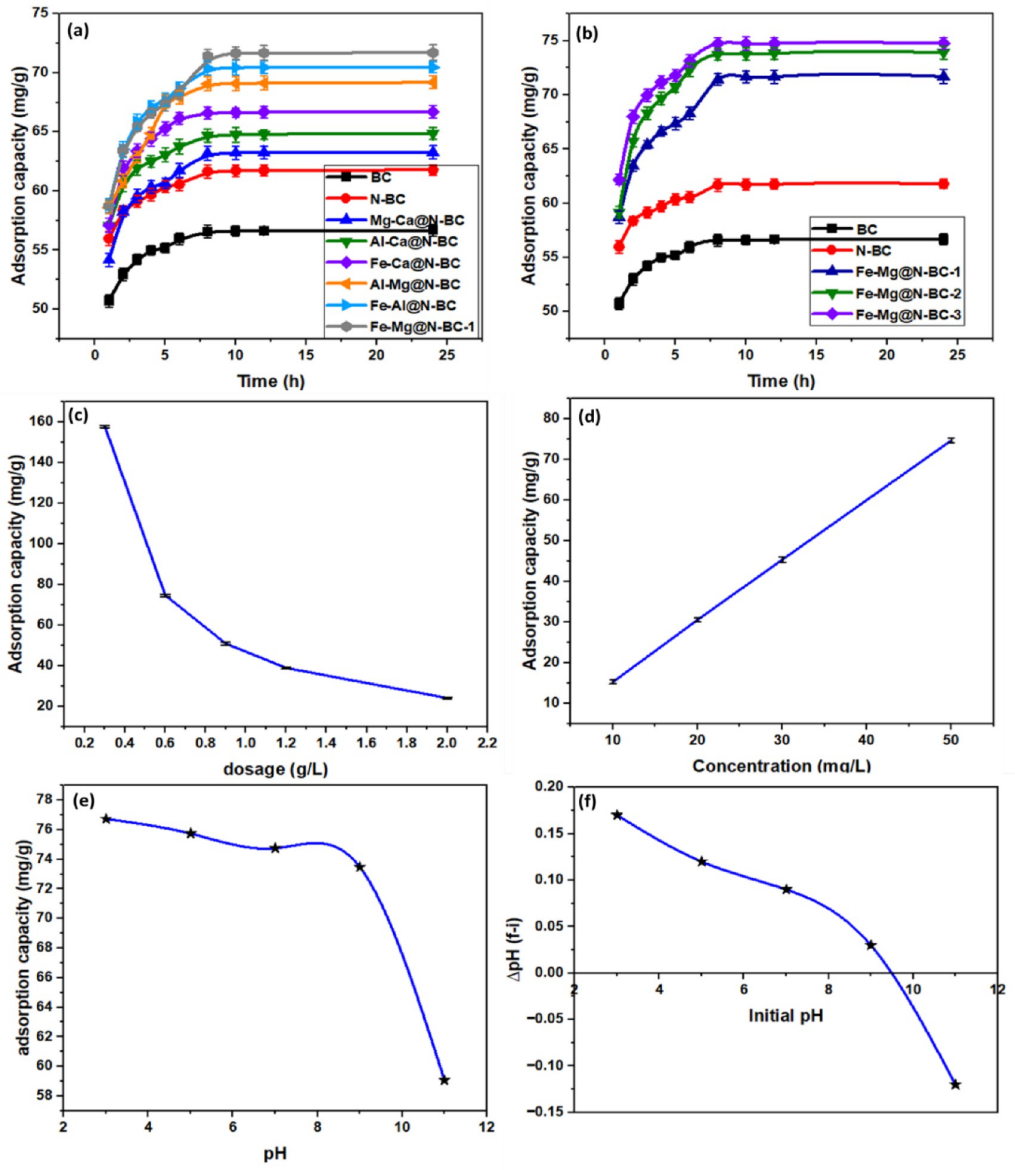
Adsorption of PCM on Fe–Mg@N-BC-3 at different (a) contact time, (b) Fe loading percentage, (c) Fe–Mg@N-BC-3 loading, (d) PCM concentration, (e) pH, and (f) pH_pzc_ of Fe–Mg@N-BC-3. Error bars represent standard deviation (SD).

Figure 4b shows the effect of Fe loading ratio on the performance of Fe–Mg@N-BC for PCM removal. Typically, increasing the Fe content from Fe–Mg@N-BC-1 to Fe–Mg@N-BC-3 enhanced the contaminant adsorption efficiency. The results show that increasing the Fe loading on the BC matrix improved the adsorption capacity from 71.8 to 73.9 mg/g and 74.7 mg/g for Fe–Mg@N-BC-1, Fe–Mg@N-BC-2, and Fe–Mg@N-BC-3, respectively. This performance improvement is ascribed to the proliferation of active surface sites facilitated by Fe, Mg, and N, which augmented the interfacial interactions between the PCM and Fe–Mg@N-BC-3. Mei^46^ et al. indicated that increasing the load of Fe species onto the BC matrix can increase its specific surface area and promote the formation of a porous structure. Furthermore, the cointroduction of Fe and N onto the BC matrix can produce more graphite carbon, change the surface charge, increase the polarity, introduce N-containing groups, and promote Fe reduction to enhance the magnetism of the BC. This modification consequently provides a greater abundance of active adsorption sites for the removal of the PCM from water. Furthermore, an increased loading ratio contributed to the modification of the surface architecture and an increase in electron density surrounding the active sites, thereby positively influencing the bonding strength with PCM molecules and, consequently, the overall removal efficacy. The adsorption performance of Fe–Mg@N-BC-3 was compared to that of recently reported adsorbents. As summarized in Table 1, Fe–Mg@N-BC-3 exhibits remarkable adsorption capacity (157.9 mg/g), outperforming functionalized, N-doped, and Me-modified biochars, while also offering advantages in cost-effectiveness and sustainable production. This superior performance can be attributed to the synergistic effects of Fe/Mg doping and N functionalization, which enhance the surface area, porosity, and availability of active sites. The comparison clearly demonstrates the effectiveness of Fe–Mg@N-BC-3 relative to other BCs under similar experimental conditions.

**Table 1:** Comparative Analysis of Adsorption Performance of BC-Based Adsorbents for Removal of PCM

Adsorbent	Type of adsorbent	q_m_ (mg/g)	Notable conditions mentioned in the study	Reference
Activated BC	municipal solid wastes	24.10	C_o_ = 50 mg/L, dose = 20 mg, time = 24 h, *T* = 30 °C.	^47^
BC	Citrus Waste	45.00	C_o_ = 25 mg/L, dose = 0.2 g/L, time = 24 h, *T* = 65 °C, agitation = 200 rpm.	^48^
BC	Quercus Brantii (Oak) acorn	45.45	pH = 3, C_o_ = 100 mg/L, dose = 1 g/L, time = 150 min.	^12^
Activated BC	*Ailanthus altissima* leaves	75.60	C_o_ = 50 μg/mL, time = 60 min, *T* = 23 °C, agitation = 500 rpm.	^49^
Poly(*N*-isopropylacrylamide) modified graphene oxide	flake graphite	107.40	pH = 7.0, dose = 0.1 mg/mL, time = 8h, *T* = 20 °C.	^50^
N-doped BC activated with H_3_PO_4_	Microalgae biomass	120.70	pH = 6, C_o_ = 150 mg/L, dose = 2.0 g/L, *T* = 23 °C, time = 4 h.	^51^
Functionalized BC	bamboo sawdust	125.31	pH = 6.8, C_o_ = 20 mg/L, dose = 0.5 g/L, time = 120 min.	^52^
MgO/Al_2_O_3_-modified BC	rice husk	137.40	pH = 8, C_o_ = 10 mg/L, dose = 0.1 g, time = 8 h, *T* = 40 °C, agitation = 100 rpm.	^53^
Fe–Mg@N-BC-3	Coconut shell	157.90	pH = 7, C_o_ = 50 mg/L, dose = 0.3 g/L, time = 8 h, *T* = 45 °C, agitation = 200 rpm.	This work
Acid-treated multiwalled carbon nanotubes	-	160.00	C_o_ = 10 mg/L, dose = 1.0 g/L, *T* = 25 °C, agitation = 170 rpm.	^54^

#### 3.2.2 Effects of PCM Concentration and Fe–Mg@N-BC-3 Loading

As shown in Figure 4c, the adsorption capacity was slightly lower when Fe–Mg@N-BC-3 loading increased from 0.3 to 2 g/L. This behavior is attributed to the presence of abundant active sites relative to the diminished PCM concentration.^55^ The highest adsorption capacity (157.9 mg/g) was achieved at an adsorbent dosage of 0.3 g/L due to the optimal utilization of the active sites. The effect of Fe content on the adsorption capacity was also studied, and increasing Fe loading initially enhanced adsorption capacity by providing more active sites. However, excessive Fe loading may cause aggregation of Fe species and pore blockage, thereby reducing available sites and overall adsorption performance.^56^ However, a dosage of 0.6 g/L was selected for subsequent adsorption studies, as it provides a better balance between adsorption capacity and removal efficiency, making it more suitable for practical applications. Meanwhile, increasing the PCM concentration from 10 to 50 mg/L at a constant Fe–Mg@N-BC-3 loading of 0.6 g/L increased the adsorption capacity from 15.2 to 74.7 mg/g (Figure 4d) due to a higher number of PCM molecules in solution for adsorption. As the PCM concentration increases at constant adsorbent loading, the active sites will eventually be fully occupied, and no further adsorption will take place.^57^

#### 3.2.3 Influence of pH

The adsorption performance of an adsorbent is strongly influenced by solution pH, which governs both the speciation of the target contaminant and the surface charge of the adsorbent. As shown in Figure 4e, a consistently high adsorption capacity of ∼73.5–76.7 mg/g was maintained throughout the evaluated pH range between pH 3 and 9 but decreased drastically to 59 mg/g at pH 11. This observation can be explained based on the electrostatic interaction between the adsorbent surface and PCM. The point of zero charge (pH_pzc_) for the Fe–Mg@N-BC-3 (Figure 4f) was determined to be 9.5, signifying a net positive surface charge below this threshold and a net negative charge above it.^58^ Meanwhile, the primary acid dissociation constant (p*K*_a_) of PCM is 9.38,^59^ indicating its predominant existence as a neutral molecule at pH levels below 9.38, transitioning to a negatively charged anion at higher pH values. Hence, as the pH rises above the PZC, electrostatic repulsion between the negatively charged surface and the PCM resulted in reduced adsorption capacity.^53^ Collectively, these results demonstrate that electrostatic interactions play a dominant role in the adsorption of PCM onto Fe–Mg@N-BC-3, with pH-dependent changes in both adsorbent surface charge and contaminant speciation governing the overall removal efficiency.

#### 3.2.4 Adsorption Kinetics Study

High adsorption capacity and rapid kinetics are essential properties of effective adsorbents used for water treatment and pollutant removal.^60^ The nonlinearized forms of the pseudo-second-order PFO, PSO, Elovich, and IPD equations were employed to study the kinetics of the adsorption. Typically, the adsorption rate and equilibrium uptake followed the ascending order BC Figure 5a–b < N-BC Figure 5c–d < Fe–Mg@N-BC-3 Figure 5e–f, confirming that N and bimetallic Fe/Mg incorporation accelerated the adsorption process by increasing the density of effective binding sites. Comparison of the correlation coefficients and root-mean-square errors (Table S2) between the kinetic models revealed that the nonlinear PSO model (R^2^ = 0.993, RMSE = 0.294) provided the most consistent description of the kinetic profiles, indicating that the overall rate is governed by site availability and surface interaction strength rather than by a simple first-order process. This observation suggests that chemisorption is the primary rate-limiting mechanism governing the adsorption process.

**Figure 5:**
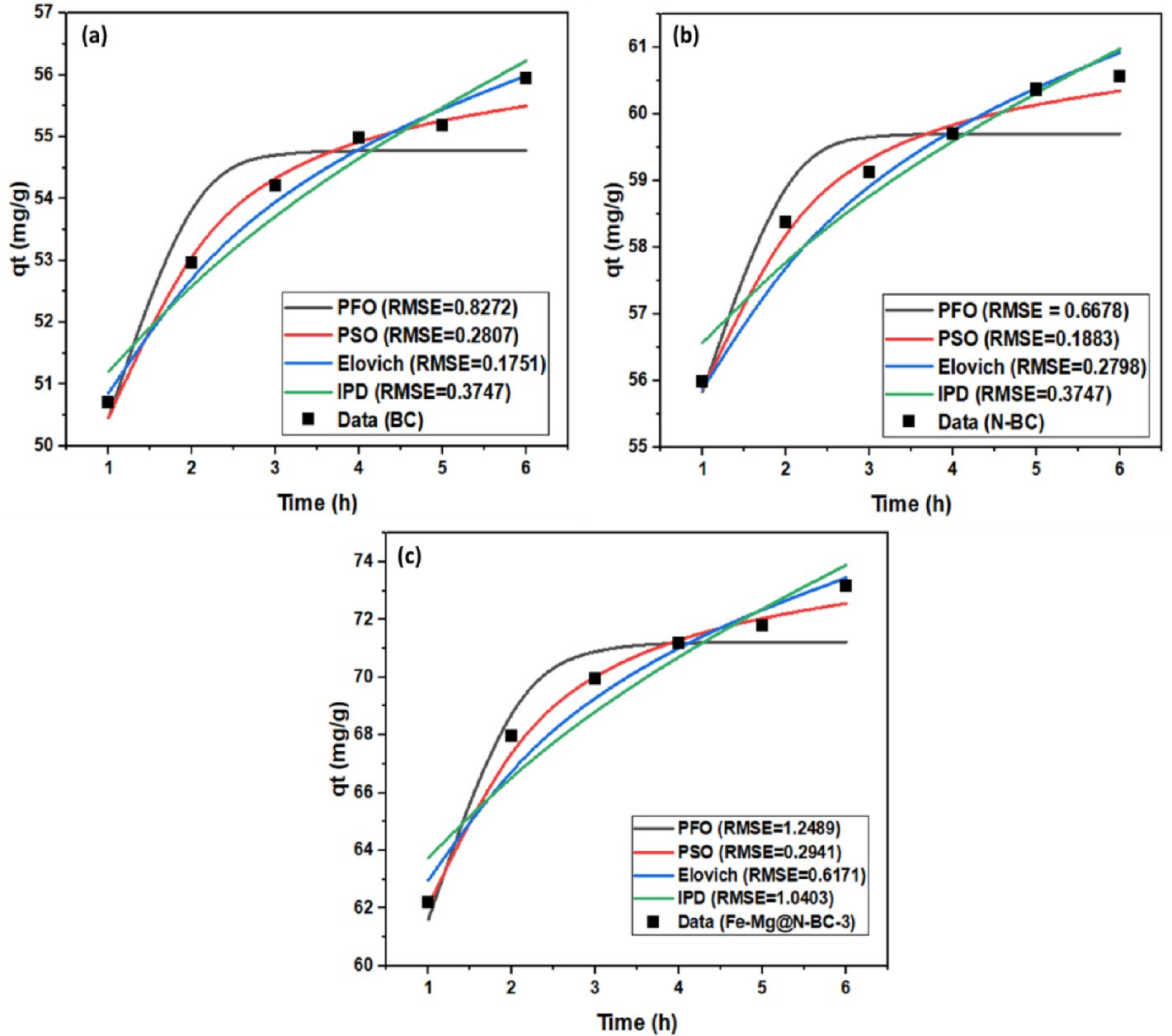
Kinetic equations fitting for PCM adsorption on (a) BC, (b) N-BC, and (c) Fe–Mg@N-BC-3. (Adsorbent loading = 0.6 g/L, PCM concentration = 50 mg/L, pH = 7, shaking speed = 200 rpm, and contact time = 8 h).

Kinetic model comparison showed that the adsorption data were best described by the PSO model, which gave the highest value of R^2^ = 0.999 compared to the other models. The model equilibrium capacity (q_e_) was calculated using an equation in Table S2 to be 75.1 mg/g, which is close to the practical value for adsorption capacity (74.7 mg/g), supporting the suitability of PSO and indicating that the PSO model better describes the adsorption behavior than other models. These results demonstrate that the superior performance of Fe–Mg@N-BC-3 arises from synergistically enhanced surface reactivity together with improved mass transfer facilitated by its developed pore structure.

#### 3.2.5 Isotherms and Thermodynamics

The equilibrium adsorption data were fitted to Langmuir, Freundlich, and Redlich–Peterson isotherm models Figure 6 (Table S3). Generally, the Langmuir model produced remarkable fit with R^2^ ≥ 0.99 for all three adsorbents, and the calculated RL values all fell within the range of 0 to 1, confirming that the adsorption process was favorable under the investigated conditions.^61^ The Freundlich isotherm, with a correlation coefficient R^2^ > 0.97, provided parameters indicative of surface heterogeneity. Also, 0 < 1/*n* < 1, which is considered favorable for adsorption.^61^ The Redlich–Peterson model, which incorporates features of both Langmuir and Freundlich isotherms, was also applied. The β was determined to be 0.99 for BC, 0.87 for N-BC, and 0.95 for Fe–Mg@N-BC-3, values close to unity, indicating that the adsorption behavior strongly approximates a Langmuir-type isotherm.^62–64^ This observation suggests that the adsorption process potentially involves the monolayer formation of chemical bonds between metals and the PCM.

**Figure 6:**
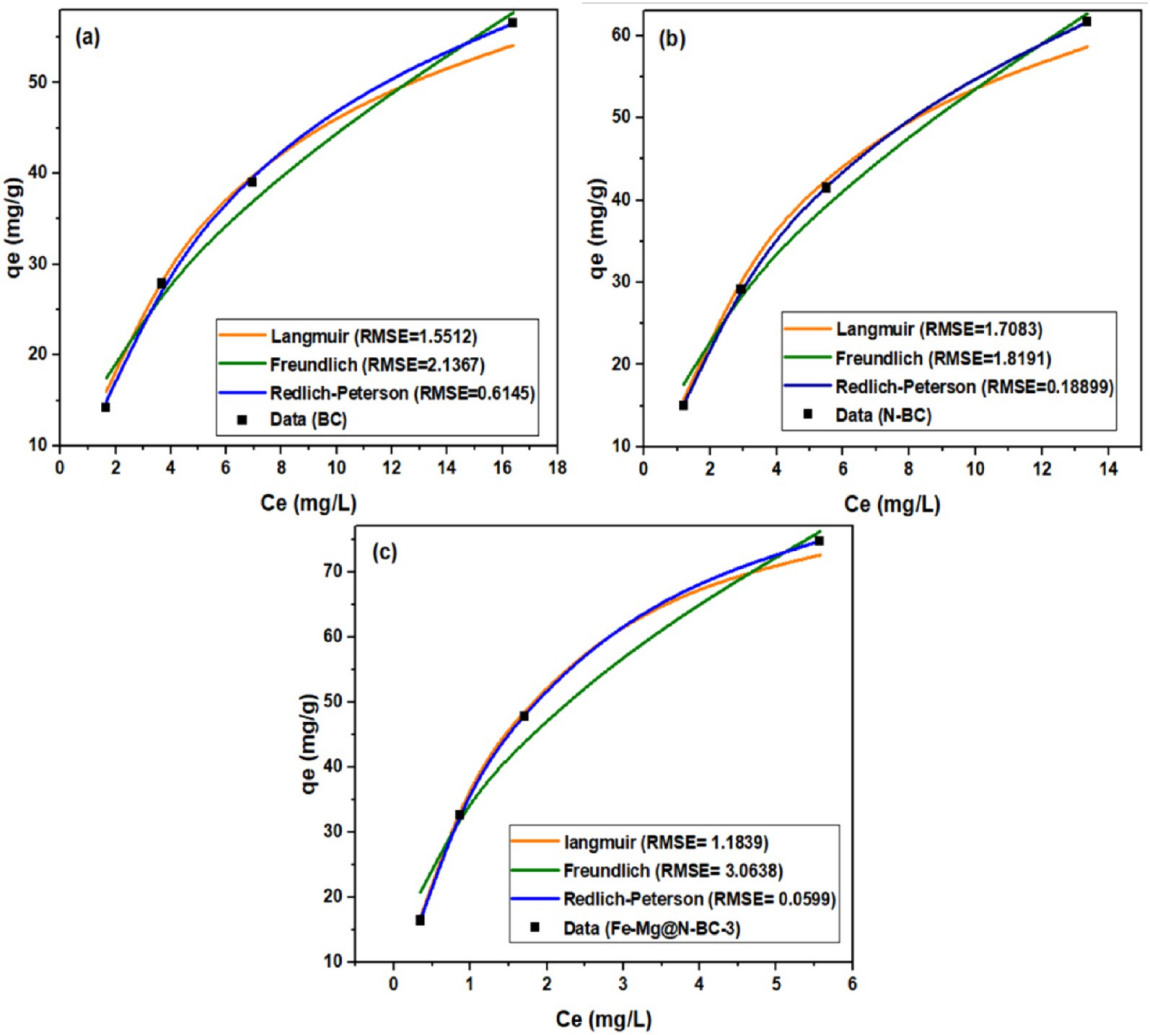
Adsorption isotherm model fitting for PCM on (a) BC, (b) N-BC, and (c) Fe–Mg@N-BC-3 at 25 °C. (Adsorbent loading = 0.6 g/L, PCM concentration = 50 mg/L, pH: = 7, shaking speed = 200 rpm, equilibrium time = 8 h).

The thermodynamic parameters for the adsorption of PCM onto BC, N-BC, and Fe–Mg@N-BC-3 were evaluated to elucidate the nature of the adsorption process. The Gibbs free energy values (ΔG°) calculated using the following equation: ΔG^o^ = – RTlnK_d_ (where R is the gas constant and K_d_ is the equilibrium constant) were negative across the studied temperature range (25–45 °C). The ΔG° becomes more negative with increasing temperature and, upon multielement incorporation (Fe–Mg@N-BC-3), more negative than N-BC and BC (Table S4), indicating a spontaneous adsorption phenomenon in all adsorbents. This confirms that the bimetallic incorporation and N doping increase adsorption favorability. The negative ΔG° values and their increase with temperature are consistent with an endothermic process, a conclusion further supported by the observed rise in equilibrium adsorption capacity with temperature from 25 °C (RE% = 88.95), 35 °C (RE% = 94.88), to 45 °C (RE% = 97.99). Similarly, the changes in enthalpy (ΔH°) and entropy (ΔS°) were calculated (Tables S2 and S5) and the positive results confirm the endothermic nature of the adsorption and the increase in randomness at the solid-solution interface during adsorption, respectively, due to the favorable immobilization of PCM molecules onto the Fe–Mg@N-BC-3 surface.^53,65^ These results are consistent with thermally enhanced adsorption behavior and indicate strong interactions between the surface and adsorbed molecules.

### 3.3 Assessment of Water Matrix Effects

Competing ions and complex matrices in real water samples remain major challenges for the practical application of adsorbents.^66^ The influence of ionic strength (10–50 mM KCl) as well as cations (Ca^2+^, Na^+^, Mg^2+^, Fe^3+^) and anions (Cl^–^, CO_3_^2–^, HCO_3_^–^, SO_4_^2–^) on PCM adsorption was evaluated to simulate the complexity of natural water sources, and the results are presented in Figure 7. The anions and cations were evaluated at concentrations of 0, 0.1, 1.0, and 10.0 mM. Typically, ionic strengths in the range of 10–50 mM (KCl) do not show a clear effect on the adsorption process, with the removal efficiency remaining at relatively high levels (Figure 7c). Meanwhile, the presence of anions (Cl^–^, CO_3_^2–^, HCO_3_^–^, SO_4_^2^) or cations (Ca^2+^, Na^+^, Mg^2+^, Fe^3+^) also had very little effect on PCM adsorption. At 0.1 mM, the observed removal ranged from 87.8 ± 0.6% to 88.8 ± 0.4%, while at 1 mM, it varied from 86.5 ± 0.6% to 88.2 ± 0.4%, except for the highest concentration (10 mM), a decrease in removal efficiency was observed, with values ranging between 84.2 ± 0.3% and 87.9 ± 0.3%. Statistical analysis (ANOVA) showed that this change was statistically significant (*P* < 0.05), with PCM removal efficiency remaining at relatively high levels.

**Figure 7:**
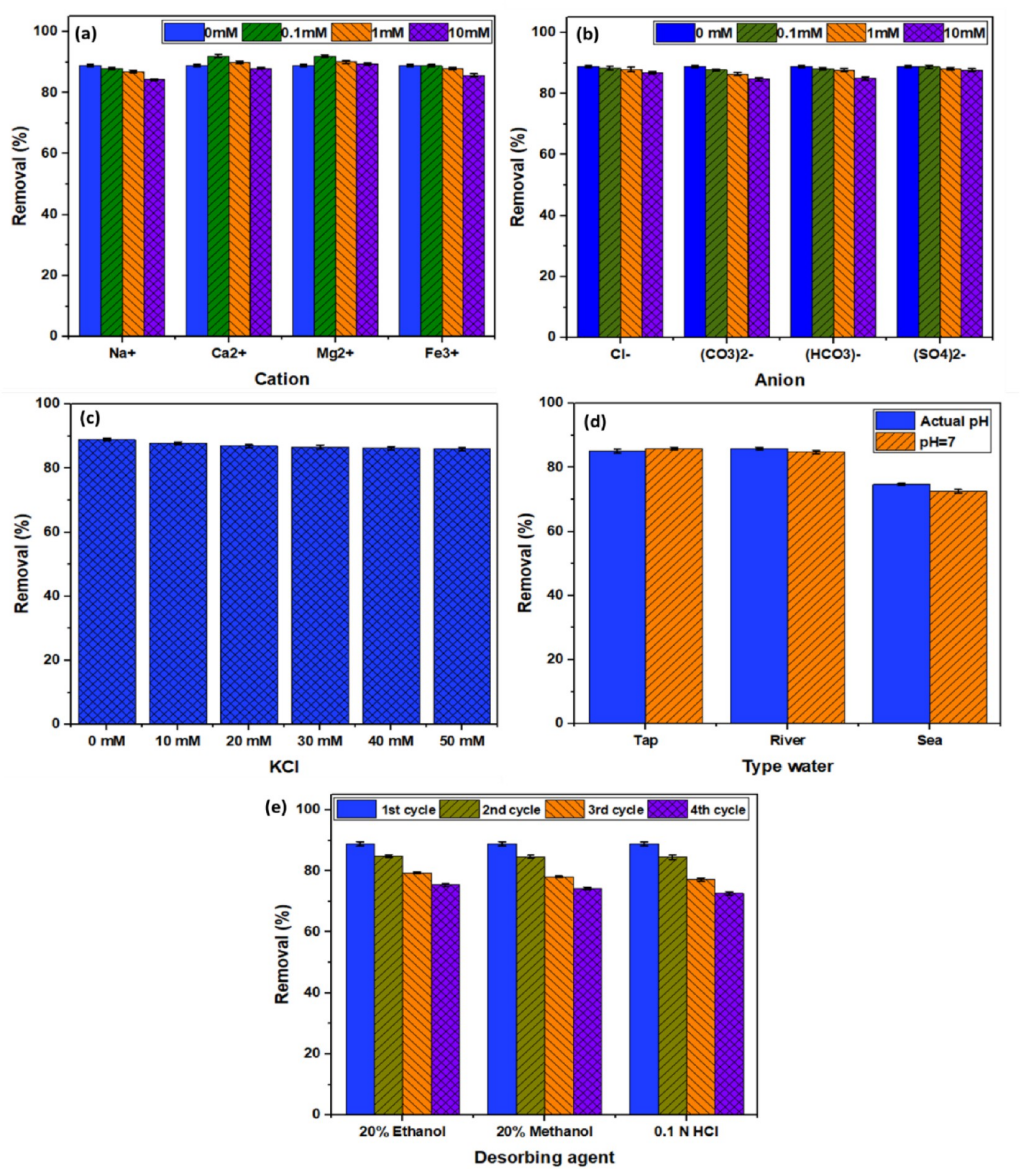
Effects of (a) cations (Ca^2+^, Na^+^, Mg^2+^, and Fe^3+^), (b) anions (Cl^–^, CO_3_^2–^, HCO_3_^–^, and SO_4_^2–^), (c) ionic strengths, (d) real water matrices (tap, river, and seawater), and (e) regeneration agents on PCM adsorption. Error bars represent the standard deviation (SD).

For cations (Ca^2+^, Na^+^, Mg^2+^, and Fe^3+^),. all showed statistically significant differences (*P* < 0.05) across different concentrations. For anions, CO_3_^2–^ and HCO_3_^–^ both showed statistically significant changes (*P* < 0.05), while the changes for Cl^–^ and SO_4_^2^ were not statistically significant (*P* > 0.05), indicating that the effect is ion-dependent. Although these effects remain relatively limited, CO_3_^2–^ showed the highest competitive effect among the anions, possibly due to the effect of ionic strength, competitive adsorption, and complex formation,^25^ leading to reduced uptake of PCM. Meanwhile, Mg^2+^/Ca^2+^ had the greatest positive effect among the cations on PCM adsorption. The positive effect of Mg^2+^/Ca^2+^ enhancing adsorption can be explained in divalent cations predominantly through the mechanism of cation bridging.^67^ Overall, Fe–Mg@N-BC-3 proves to be a promising adsorbent, capable of operating effectively in real waters containing a wide range of competing ions.

### 3.4 Performance in Real Water

The performance of Fe–Mg@N-BC-3 as an adsorbent for PCM removal in tap, river, and seawater at pH 7 and actual pH was evaluated, and the results are presented in Figure 7d. In tap water, the PCM removal efficiency was slightly higher at pH 7 (85.9 ± 0.8%) than at the actual pH of 8.43 (85.2 ± 0.6%). In river water, the removal of PCM was slightly higher at the actual pH of 6.31 (85.9 ± 0.4%) than at pH 7 (84.9 ± 0.5%). Also, in seawater, the removal of PCM was slightly higher at the actual pH of 6.54 (74.7 ± 0.4%) than at the adjusted pH of 7 (72.6 ± 0.2%). This is attributed to weaker matrix interference in tap water compared to river water and seawater. Typically, tap water is characterized by reduced concentrations of natural organic matter and lower ionic strength, which mitigates pore blockage and the competitive occupancy of adsorption sites. In contrast, river water contains dissolved organic matter^68^ which can foul the adsorbent surface and compete for active sites, reducing the accessible porosity and adsorption efficiency. Meanwhile, seawater exhibits very high ionic strength and abundant inorganic ions, which intensify competitive adsorption and can promote particle aggregation and site shielding, leading to the lowest PCM removal. These results indicate that Fe–Mg@N-BC-3 exhibits good performance and acceptable PCM removal efficiency when applied to real tap, river, and seawater samples.

### 3.5 Regeneration/Desorption Study and Stability of the Adsorbent

The regeneration of the Fe–Mg@N-BC-3 was performed using solvents such as ethanol, methanol, and HCl (Figure 7e). The solvents can desorb the PCM while preserving the material’s structural and chemical composition. It is evident that HCl, ethanol, and methanol are effective in regenerating the adsorption efficiency of PCM, with a slight decrease in performance observed during the fourth regeneration cycle. During the first cycle, the sorbent achieved a removal of PCM at 88.9 ± 0.7%. When a 20% ethanol solution was used, PCM removal efficiencies of 84.9 ± 0.5%, 79.4 ± 0.2%, and 75.5 ± 0.5% were recorded during the second, third, and fourth cycles, respectively. Similar results were obtained when using a 20% methanol solution (second cycle = 84.6 ± 0.5%, third cycle = 78.1 ± 0.2%%, and fourth cycle = 74.2 ± 0.5%). The PCM removal using 0.1 N hydrochloric acid achieved 84.5 ± 0.6%, 77.1 ± 0.6%, and 72.6 ± 0.6%, in the next three cycles. The decrease in PCM adsorption efficiency during the fourth regeneration cycle can be ascribed to (1) chemisorption involving the formation of stable surface complexes or precipitates on the active sites, and (2) pore blockage, resulting in a gradual loss of functionality.^69^

In comparison, ethanol showed slightly higher PCM removal, followed by methanol, while hydrochloric acid showed slightly lower values. This can be explained by the solubility of PCM in polar organic solvents, the lower polarity of the solvent, and the disruption of hydrogen bonds between Fe–Mg@N-BC-3 molecules and PCM, which enhance the solubility of polar groups and the aromatic ring.^70^ However, statistical analysis (ANOVA) showed that the differences among the three solvents were not statistically significant (P = 0.840), confirming that ethanol, methanol, and hydrochloric acid are equally effective in PCM regeneration. This implies that these solvents can be effectively used to regenerate spent adsorbent. Such regeneration approaches using alcohols (e.g., ethanol and methanol) and acidic solutions (e.g., HCl)^53,71^ are widely reported for BC-based adsorbents, as they facilitate desorption by disrupting adsorbate–adsorbent interactions without the need for high-energy thermal treatment.

As illustrated in Figure S1, Fe leaching was quantified using atomic absorption spectroscopy. After the adsorption cycle, the supernatant was filtered and then analyzed to quantify the concentrations of Mg^2+^ and Fe^3+^. The measured Fe^3+^ leaching concentrations ranged from 0.12 to 0.48 mg/L, while Mg^2+^ leaching concentrations were lower than 0.53 mg/L. These concentrations are well below drinking-water guideline limits according to the National Standard for Drinking Water Quality—NSDWQ (prepared by the Engineering Services Department, Ministry of Health Malaysia), where the maximum acceptable values for Fe and Mg are 1 and 150 mg/L, respectively. Therefore, the leached Mg or Fe from Fe–Mg@N-BC-3 is not of major concern according to the obtained results, confirming the high structural stability of Fe–Mg–N-BC-3.

### 3.6 Possible Mechanism of PCM Adsorption Onto Fe–Mg@N-BC-3

The overall adsorption process can be described by three main stages: (1) diffusion of PCM molecules through the external boundary liquid layer, (2) adsorption onto active sites on the BC surface, and (3) intraparticle diffusion into pores and internal adsorption sites.^72^ The involvement of surface functional groups and metal-related active sites in these stages can be evidenced by changes in the chemical bonding environment of the adsorbent after adsorption. Figure 8a shows the FTIR spectrum of Fe–Mg–N-BC-3 after PCM adsorption, indicating that after PCM adsorption, new peaks or increased intensity in the range of 595–655 cm^–1^ associated with M–O bending vibrations (such as Fe–O, Mg–O) emerged. This observation indicates interactions between PCM’s hydroxyl/carbonyl groups, and the metal species supported on the BC. A shift from 1525 to 1529 cm^–1^ (C=C or N–H bending) indicates that the amide group (—NH—C=O) of PCM interacted with the grafted N sites, likely via π-π interaction with carbon layers and/or hydrogen bonding. The band at 1700 cm^–1^, attributed to C=O stretching (carbonyl group of PCM), confirms the presence of PCM on the adsorbent surface. The slight shift in this peak position suggests a change in hydrogen bonding environment after adsorption. The band at 2360 cm^–1^, corresponding to adsorbed CO_2_, remains present. Changes in intensity and position of the 3500–3935 cm^–1^ band suggest that hydroxyl groups on N-enriched BC participated in strong hydrogen bonding with PCM.

**Figure 8:**
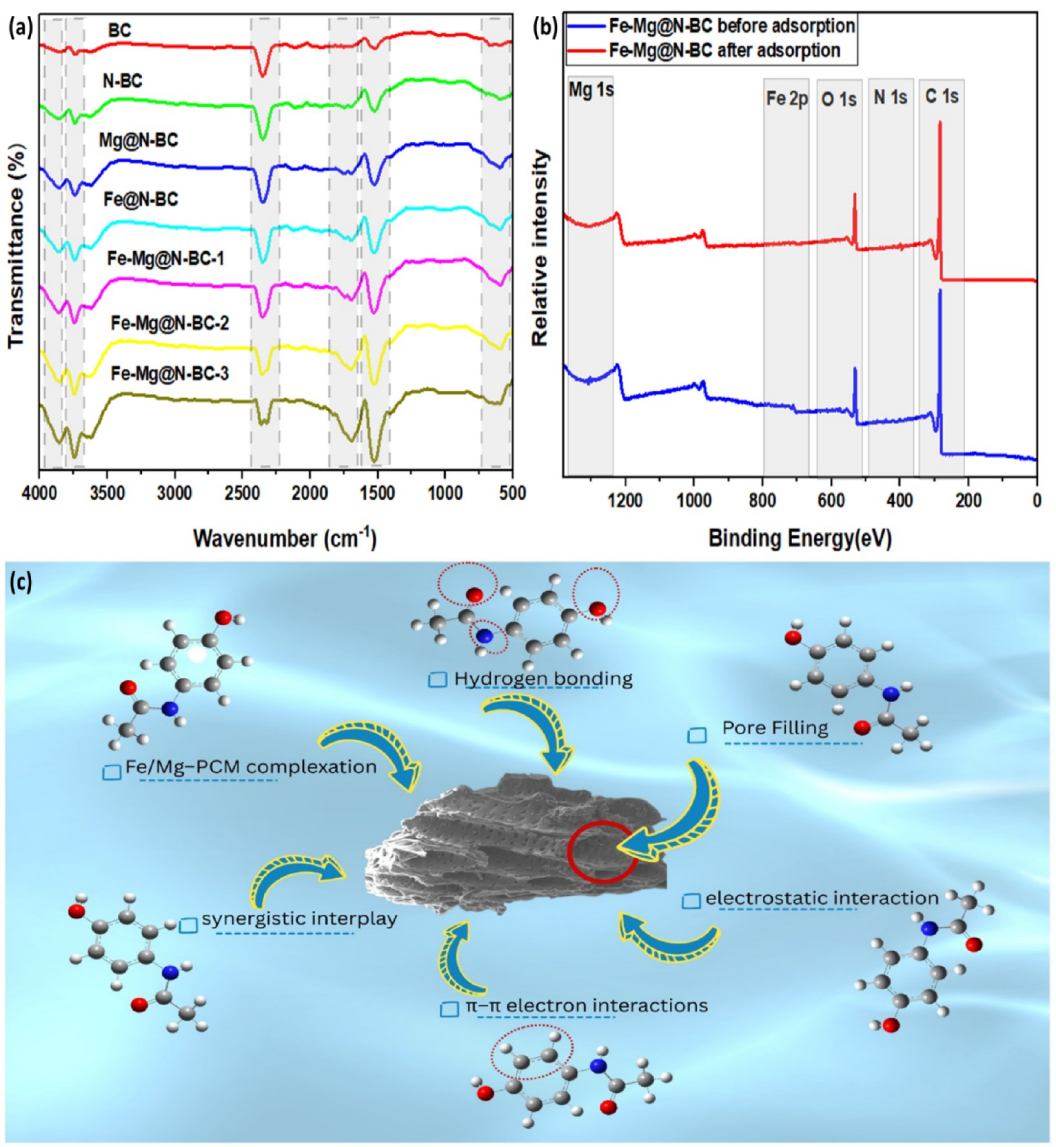
(a) FTIR spectra of adsorbents after the adsorption of PCM, (b) XPS survey spectra of Fe–Mg@N-BC-3 before and after adsorption, and (c) the possible mechanism of PCM adsorption onto Fe–Mg@N-BC-3.

To further investigate the changes in surface chemistry of the adsorbent, XPS spectra of Fe–Mg@N-BC-3 before and after adsorption were obtained. The survey scans and chemical composition comparison of C, N, O, Fe, and Mg for Fe–Mg@N-BC-3 are shown in Figure 8b and Tables S5–S6. In the pristine Fe–Mg@N-BC-3, the C 1s spectrum (Figure S2) can be deconvoluted into metal carbide C-M (∼283 eV),^73^ C–C/C=C (∼284.8 eV), and C–O/C–N (∼286 eV).^74^ The N 1s spectrum can be deconvoluted into pyridinic N (∼398.6 eV), pyrrolic N (∼400.1 eV), N-oxide (∼402 eV),^31^ and π-π* (∼404 eV).^75^ The O 1s spectrum was deconvoluted into metal oxides (∼530 eV), hydroxyl C–O (∼531.7 eV),^37^ and esters/anhydrides C–O–C (∼533.8 eV).^76^ The Fe 2p spectrum showed Fe^2+^ 2p_3/2_ (∼707 eV), Fe^3+^ 2p_3/2_ (∼711 eV), satellite peaks (∼716.5 eV),^77^ and corresponding Fe^2+^/Fe^3+^ 2p_1/2_ peaks (∼721 and ∼724.8 eV).^31^ The Mg 1s spectrum displayed Mg metal (∼1301.5 eV) and Mg oxides (∼1304 eV).^78^ Overall, the XPS results confirm successful metal incorporation and N doping into the BC.

After adsorption, the Mg peak disappeared, likely due to surface coverage by a PCM layer and/or interaction with organic functional groups, reducing its apparent surface concentration within the XPS analysis depth. Meanwhile, the C–C/C=C (graphitic) component decreased from 83.14 to 75.24 at. % after adsorption, suggesting coverage of the carbon surface by a PCM layer and reduced substrate electron detection. This suggests π-π interactions between the aromatic PCM and the carbon structure.^79^ These π-based interactions include cation-π interactions between metal cations and aromatic moieties driven by electrostatic attraction, anion-π interactions involving electron-deficient aromatic rings, π-π donor–acceptor interactions between two π-systems, as well as n-π interactions arising from lone-pair electrons interacting with aromatic π-systems.^80^ Conversely, the C–O/C–N component increased from 12.77 to 24.76 at. % after adsorption, consistent with phenolic/amide groups in PCM and the formation of new surface interactions (hydrogen bonding, π-π stacking, and coordination). The metal carbide signal (∼283 eV) also disappeared, likely also due to surface coverage and/or chemical transformation. In the N 1s spectrum, pyridinic N and N-oxide decreased, while pyrrolic N increased after adsorption, indicating electron redistribution due to π-π interactions and hydrogen bonding, as well as surface interaction with organic adsorbates and possible oxidation/protonation of N sites.^81^ The incorporation of N functionalities enhances surface polarity and furnishes specific sites for hydrogen bonding with hydroxyl and amide groups on the adsorbate. The presence of pyridinic N (∼398.6 eV) also suggests Fe–N_*x*_ coordination (Fe–N_*x*_ active sites).^82^ Moreover, Fe–N_*x*_ active sites function as metal–ligand coordination centers, enabling chemisorption via complexation with oxygen atoms present in the PCM. Although Figure 8b shows minimal changes in the XPS spectra of Fe–Mg@N-BC-3 after PCM adsorption, suggesting that the overall surface chemical states remain largely unchanged, the adsorption process can still involve weak interactions such as Me-PCM complexation or localized chemisorption. These subtle interactions may not produce pronounced shifts in the XPS spectra despite contributing to the observed adsorption behavior.

The O 1s spectrum shows a decrease in C–O and metal oxide signals and an increase in C–O–C after adsorption, possibly attributed to (i) hydrogen bonding between PCM and surface functional groups, and (ii) the formation of M–O–C bridges with phenolic oxygen. This suggests that adsorption is driven by hydrogen bonding and phenolic interactions with active centers.^79^ Meanwhile, the Fe 2p analysis revealed a relative increase in Fe^2+^ and a decrease in Fe^3+^ after adsorption, indicating electron transfer at the carbon–metal/PCM interface and partial reduction of Fe^3+^ to Fe^2+^. This behavior aligns with reported activation mechanisms in Fe-doped BC.^80^ Based on the results, it can be deduced that the PCM adsorption on Fe–Mg@N-BC-3 is attributed to a combination of metal complexation, cation-π interactions, hydrogen bonding, and π-π stacking. The possible adsorption pathways are illustrated in Figure 8c.

### 3.7 Density Functional Theory (DFT) Calculations

Density functional theory (DFT) calculations were performed to gain atomic-level insight into the adsorption behavior of PCM using BC, Mg@N-BC, Fe@N-BC, and Fe–Mg@N-BC-3 surfaces and to identify the dominant adsorption sites and interaction mechanisms. As shown in Figure S3a, the optimized molecular geometry of PCM contains multiple electron-donating and electron-accepting functional groups, including a phenyl ring, amide, and hydroxyl groups, rendering it highly reactive to surface-mediated adsorption via hydrogen bonding, π-π interactions, and coordination with metal ions. Frontier molecular orbital analysis reveals that the HOMO of PCM (Figure S3b) is predominantly distributed over the aromatic ring, phenyl moiety, and phenolic oxygen, indicating that these regions act as the primary electron-donating sites during adsorption. The high π-electron density on the aromatic ring favors π-π interactions with carbonaceous surfaces, thereby increasing adsorption energy.^83^ In addition, the elevated electron density on the phenolic −OH group facilitates hydrogen bonding and donor–acceptor interactions with heteroatoms and metal-doped active sites,^84^ contributing to the enhanced affinity of PCM toward N-doped and Fe–N-based BC materials. Conversely, the LUMO (Figure S3c) is mainly localized on the amide group, particularly the amide N and carbonyl oxygen, suggesting that these moieties function as the primary electron-accepting sites. This electronic feature favors charge-transfer interactions and metal-assisted surface complexation^85^ with Fe-containing and N-doped carbon surfaces, thereby enhancing adsorption strength and stability.

A hexagonal graphite-like unit cell (space group *P*6/*mmm*) was used to construct the pristine biochar (BC) model. After geometry optimization, the lattice parameters converged to a = b = c = 20.0 Å, with α = β = 90° and γ = 120°, yielding a unit-cell volume of 6928.20 Å^3^ and a density of 0.29 g cm^–3^. Negligible residual forces and near-zero external pressure confirmed structural stability. The dispersion-corrected total energies were −2838.69 eV for BC, −4492.75 eV for Fe@N-BC, and −4689.07 eV for Mg@N-BC. Following PCM adsorption, total energies decreased significantly, indicating enhanced structural stability due to charge transfer, electron redistribution, and coordination interactions between PCM functional groups and Me-N active sites. Shortened adsorption distances after optimization further confirm relatively strong molecule–surface interactions. Adsorption locator calculations show that doping markedly improves adsorption affinity. Pristine BC exhibited an adsorption energy of −49.48 kcal mol^–1^, dominated by π-π interactions, hydrogen bonding, and van der Waals forces.^86^ N doping increased surface polarity and reactivity, strengthening hydrogen bonding and electrostatic interactions with PCM. Mg–N–BC showed a higher adsorption energy (−70.43 kcal mol^–1^), where π-π donor–acceptor interactions are reinforced by hydrogen bonding, with Mg acting as a Lewis acidic site enabling metal-assisted surface complexation.^20^ Fe@N-BC exhibited stronger adsorption (−72.81 kcal mol^–1^) due to Fe–N_*x*_ coordination sites that promote metal-assisted complexation with the carbonyl and phenolic oxygen atoms of PCM,^80^ indicating a transition toward mixed physicochemical adsorption. The highest adsorption energy was obtained for Fe–Mg@N-BC-3 (−76.59 kcal mol^–1^), reflecting synergistic effects between Fe–N_*x*_ active sites and Mg-induced structural stabilization, which preserves site accessibility and enhances interfacial charge transfer.^87^

Overall, the predicted adsorption strength follows the order Fe–Mg@N-BC > Fe–N@BC > Mg@N-BC > BC, consistent with thermodynamic parameters, BET surface accessibility, and experimental adsorption capacities. These results highlight the synergistic roles of π-π interactions, N-induced electronic modulation, and metal-assisted coordination in governing PCM adsorption on BC-derived adsorbents.

## 4 Conclusions

This study successfully demonstrated the synthesis and application of Fe–Mg@N-BC-3, derived from coconut shell pyrolysis at 700 °C, for the adsorption of PCM. The adsorbent exhibited exceptional performance, achieving a maximum adsorption capacity of 157.9 mg/g for PCM at 25 °C and a removal efficiency of 97.99% at 45 °C within 8 h. This superior efficacy is attributed to the synergistic effects of the Fe, Mg, and N active sites incorporated into the BC matrix. Comprehensive characterization via FTIR, XRD, XPS, SEM, EDX, and BET analysis confirmed the successful synthesis of the composite. Results indicated the introduction of desirable surface functional groups, a robust structural morphology, and a high specific surface area, all contributing to the enhanced adsorption capability. Adsorption was found to be pH-dependent, and high removal efficiency was maintained across a broad pH range (3–9), demonstrating the material’s robustness. Furthermore, the adsorption process exhibited a preferential performance for PCM, showing no interference from competitive ions in various water matrices, including tap, river, and seawater. The adsorption kinetics were best described by the pseudo-second-order model (R^2^= 0.9987), suggesting a chemisorption-dominated process. Equilibrium isotherm data were well-fitted by the Langmuir model (R^2^= 0.9998), indicating monolayer adsorption onto a homogeneous surface. Thermodynamic studies revealed the process to be spontaneous and endothermic, with an increase in randomness at the solid-solution interface, further supporting a chemisorption mechanism. The adsorbent also demonstrated excellent stability and practical reusability, retaining high removal efficiency over three consecutive adsorption–desorption cycles. Characterization of spent samples and DFT calculations elucidated the adsorption mechanism and confirmed the critical role of Fe, Mg, and N in enhancing adsorption performance. Given its effectiveness under varied conditions, Fe–Mg@N-BC-3 presents a promising and sustainable solution for the remediation of pharmaceutical contaminants in water.

## Supplementary Material



## Notes

Reagents; supplementary tables (Tables S1–S6); adsorption isotherms, kinetics, thermodynamics, and characterization data (XPS); Figure S1, leaching study of Fe–Mg@N-BC-3; Figure S2, high-resolution XPS spectra; Figure S3, DFT analysis (PDF)
