# Supplementary material for: A Bimetal Fe/Mg Immobilized on N‑Doped Biochar for Efficient Adsorption of Paracetamol: Performance Assessment, Mechanistic Exploration, and Density Functional Theory
Source: ACS Omega. 2026 Jun 26;11(27):39943–59. doi: 10.1021/acsomega.6c01218 (PMC13382829; doi:10.1021/acsomega.6c01218)
Supplement: Supplementary file 1 [file ao6c01218_si_001.pdf]

Supporting information

A bimetal Fe/Mg immobilized on N-doped biochar for efficient adsorption of paracetamol: Performance assessment, mechanistic exploration, and density functional theory

Mohammed A. Al-Haiqi [a,b], Choon Fu Goh[c], Wen-Da Oh[a*]

[a] School of Chemical Sciences, Universiti Sains Malaysia, 11800 Penang, Malaysia

[b] Chemistry Department, College of Science, Hadhramout University, P. O. B. 50511, Mukalla, Yemen.

[c] School of Pharmaceutical Sciences, Universiti Sains Malaysia, 11800 Penang, Malaysia

*Corresponding author: E-mail address: ohwenda@usm.my (W.-D. Oh)

Text S1: Reagents

The reagents used in this study are Magnesium nitrate hexahydrate powder $Mg(NO_3)_2.6H_2O$ (ChemAR), Aluminum nitrate nonahydrate powder $Al(NO_3)_3.9H_2O$ (QReC), Calsium nitrate hexahydrate powder $Ca(NO_3)_2.6H_2O$ (QReC), Iron (III) nitrate nonahydrate powder $Fe(NO_3)_3.9H_2O$ (QReC), Urea. CH_4N_2O (Chem AR), Paracetamol. (SIGMA-ALDRICH), ethanol C_2H_5OH (Chemiz), hydrochloric acid HCl (QRëC), sodium hydroxide NaOH (Systerm®), sodium carbonate Na_2CO_3 (R&M Chemicals), sodium bicarbonate $NaHCO_3$ (R&M Chemicals), sodium chloride NaCl (Bensoden), methanol CH_3OH (Chemiz), and potassium iodide KI (Systerm®).

Table S1. Non-Linear isotherm, kinetic, and thermodynamics equations.

Model	Equation	Parameters	Ref
**Non-Linear Isotherm models**			
Langmuir	$qe = \frac{qmax\ b\ Ce}{1 + b\ Ce}$	where, qe(mg/g) is the equilibrium loading of pollutant on biochar, Ce(mg/L) is the aqueous concentration of pollutants at equilibrium, q_{max} is the maximum monolayer adsorption capacity (mg/g), b (L/mg) is in relation to the binding energy.	1
Freundlich	$qe = Kf\ Ce^{\frac{1}{n}}$	where, qe(mg/g) is the equilibrium loading of pollutant on biochar, Ce(mg/L) is the aqueous concentration of pollutants at equilibrium Kf (L/mg) is the Freundlich adsorption capacity, 1⁄n is the adsorption intensity. When $0 < 1/n < 1$, adsorption is considered favorable. Unfavorable adsorption occurs when $1/n > 1$ and is irreversible at $1/n = 1$.	1
Redlich – Peterson	$qe = \frac{K_r Ce}{1 + \alpha Ce^{\beta}}$	where Kr is the Redlich–Peterson isotherm constant in L/g, α is a constant in L/mg, and β is an exponent that ranges between 0 and 1. At low concentrations (β ~ 1), the model approaches the Langmuir isotherm,	2

		and at high concentrations (β ~ 0), it approaches the Freundlich isotherm.	
Kinetic equations			
Pseudo first order	$qt = qe\ (1 - e^{-k1\ t})$	k_1: first order rate constant (min^{-1}), q_e: adsorbate adsorbed per gram of adsorbent, q_t: adsorbate adsorbed at time ‘t’	3 4
Pseudo second order	$qt = \frac{K2\ qe^2\ t}{1 + K2\ qe\ t}$	k_2: second order rate constant (g/mg/min), q_e: adsorbate adsorbed per gram of adsorbent, q_t: adsorbate adsorbed at time ‘t’	3 4
Elovich model	$qt = \frac{1}{\beta}\ln(\alpha\beta t + 1)$	q_t: is the adsorbed amount at time t, the parameter α: is the initial adsorption rate, β: is related to extent of surface coverage and activation energy for chemisorption	3 4
Intraparticle diffusion model (IPD)	$qt = Ki\ t^{0.5} + C$	K_i: is the intraparticle diffusion rate constant C: is a constant that reflects the thickness of the boundary layer	5 4
Thermodynamics			
Van't Hoff's	$LnKd = \frac{\Delta S^\circ}{R} - \frac{\Delta H^\circ}{RT}$	Where, Kd is the distribution coefficient, R (J/mol K) is the universal gas constant whose value is 8.314 $J.mol^{-1}.K^{-1}$, and T (K) is the absolute temperature in Kelvin. ΔH° is the standard adsorption enthalpy and ΔS° is the standard adsorption entropy.	6
The thermodynamic parameter free energy	$\Delta G^\circ = -RTlnKd$	ΔG° is the thermodynamic parameter free energy	7

Table S2. Paracetamol sorption kinetic data.

**Kinetic model**		**Adsorbents**		
		**BC**	**N-BC**	**Fe-Mg@N-BC-3**
Pseudo-first-order (PFO)	k1(h^{-1})	2.5405	2.7349	2.0025
	qe(mg/g)	54.7779	59.7091	71.2295
	R^2	0.7740	0.8131	0.8789
	RMSE	0.8272	0.6678	1.2489
Pseudo-second-order (PSO)	K2(h^{-1})	0.1447	0.1666	0.0636
	qe(mg/g)	56.6284	61.3340	75.0992
	R^2	0.9740	0.9851	0.9933
	RMSE	0.2807	0.1883	0.2941
Elovich	α	55076522.3	429882122.6	102397.3401

	β	0.3494	0.3551	0.1710
	R^2	0.9899	0.9672	0.9704
	RMSE	0.1751	0.2798	0.6171
intraparticle diffusion (IPD)	Ki	3.4617	3.0428	6.9992
	C	47.7488	53.5260	56.7479
	R^2	0.9536	0.9345	0.9160
	RMSE	0.3747	0.3747	1.0403

Table S3. Sorption isotherm parameters for paracetamol on Fe-Mg@N-BC at 25 °C.

**Isotherm**		**Adsorbents**		
		**BC**	**N-BC**	**Fe-Mg@N-BC-3**
Langmuir	qm	74	80	93
	Kl	0.1663	0.2054	0.6387
	R^2	0.9900	0.9901	0.9970
	RMSE	1.5512	1.7083	1.1839
Freundlich	Kf	13.3914	16.0527	34.0883
	1/n	0.5224	0.5253	0.4681
	R^2	0.9811	0.9887	0.9797
	RMSE	2.1367	1.8191	3.0638
Redlich – Peterson	Kr	10.9974	16.7778	59.3878
	α	0.1360	0.2763	0.6726
	β	0.9933	0.8702	0.9479
	R^2	0.9984	0.9999	0.9999
	RMSE	0.6145	0.18899	0.0599

Table S4. Sorption thermodynamics parameters for adsorption paracetamol on Fe-Mg@N-BC-3, N-BC, and BC at 25, 35, and 45 °C.

**Adsorbent**	**T (K)**	**ΔG° (KJ/mol)**	**ΔS° (J/K.mol)**	**ΔH° (KJ/mol)**	**Kd**
Fe-Mg@N-BC-3	298	-6.3562	258.8980	70.7954	13.4153
	308	-8.9452			30.8775
	318	-11.5342			81.0933
N-BC	298	-3.8064	68.6612	16.6546	4.6140
	308	-4.4930			5.8689
	318	-5.1796			7.0384
BC	298	-3.2825	54.4850	12.9540	3.4552
	308	-3.8273			4.0343
	318	-4.3722			4.8299

Table S5. Elemental content from XPS analysis (survey scan) for Fe-Mg@N-BC-3.

**Chemical composition comparison of C, N, O, Fe, and Mg for Fe-Mg@N-BC-3 adsorbent before the adsorption of PCM**		
**Peak**	**Position BE (eV)**	**Atomic conc. %**
Mg 1s	1381.364	0.34
Fe 2p	711.364	3.07
O 1s	531.364	35.46
N 1s	400.364	2.07
C 1S	284.364	59.07
**Chemical composition comparison of C, N, O, Fe, and Mg for Fe-Mg@N-BC-3 adsorbent after the adsorption of PCM**		
**Peak**	**Position BE (eV)**	**Atomic conc. %**
Mg 1s	1294.339	0.00
Fe 2p	725.339	0.27
O 1s	532.339	10.68
N 1s	399.339	1.22
C 1S	284.339	87.83

Table S6. Analysis results of high resolution XPS spectra for Fe-Mg@N-BC-3. (Peak deconvolution, peak assignment, and the calculation of the relative atomic concentrations (Atomic Conc. %) of the fitted components were performed using CasaXPS.)

**Chemical composition comparison of C, N, O, Fe, and Mg for Fe-Mg@N-BC-3 adsorbent before the adsorption of PCM**		
**spectra**	**region**	**Atomic conc. %**
C 1s	M-C	4.08
	C-C/ C=C	83.14
	C–O/C–N	12.77
N 1s	pyridinic N	16.26
	pyrrolic N	60.40
	N-oxide	20.09
	π-π* excitations	3.25
O 1s	M-O	9.98
	C–O	67.60
	C-O-C	22.41
Fe 2p	Fe^{2+} $2p_{3/2}$	1.73
	Fe^{3+} $2p_{3/2}$	43.42
	satellite	30.75
	Fe^{2+} $2p_{1/2}$	6.77
	Fe^{3+} $2p_{1/2}$	17.33
Mg 1s	Mg metal	60.16
	Mg oxides	39.84
**Chemical composition comparison of C, N, O, Fe, and Mg for Fe-Mg@N-BC-3 adsorbent after the adsorption of PCM**		

C 1s	C-C/ C=C	75.24
	C–O/C–N	24.76
N 1s	pyridinic N	6.49
	pyrrolic N	88.01
	N-oxide	5.50
O 1s	M-O	2.50
	C–O	26.59
	C-O-C	70.91
Fe 2p	Fe^{2+} $2p_{3/2}$	12.43
	Fe^{3+} $2p_{3/2}$	25.41
	satellite	31.98
	Fe^{2+} $2p_{1/2}$	12.39
	$Fe^{3+}2p_{1/2}$	17.79

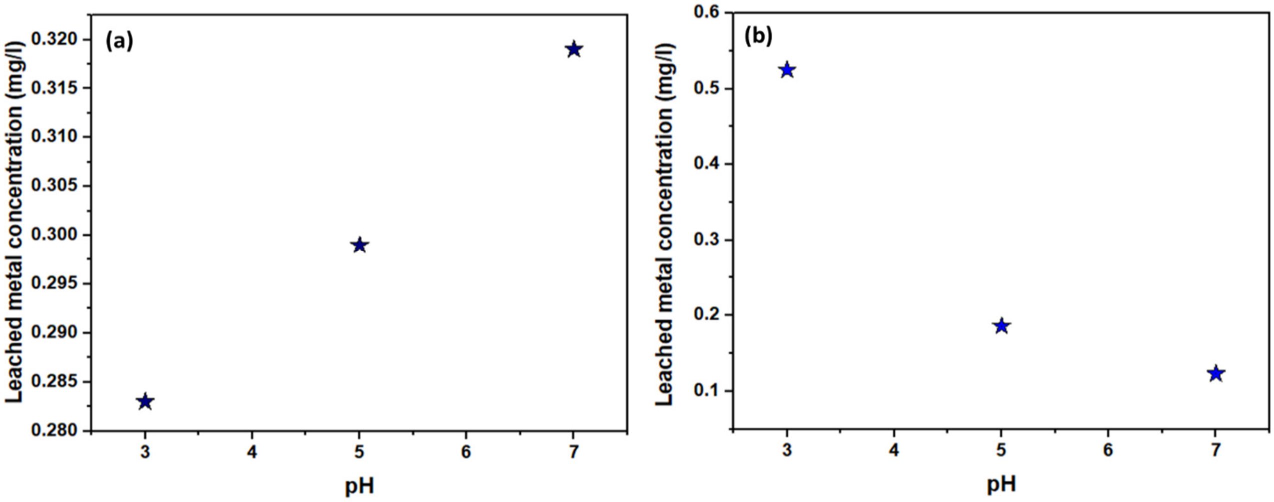

Fig. S1. Leaching study of Fe-Mg@N-BC-3 at different pH (3, 5, and 7) for (a) Fe and (b) Mg. Adsorbent loading = 2 g/L, contact time = 24 h, and agitation speed = 200 rpm.

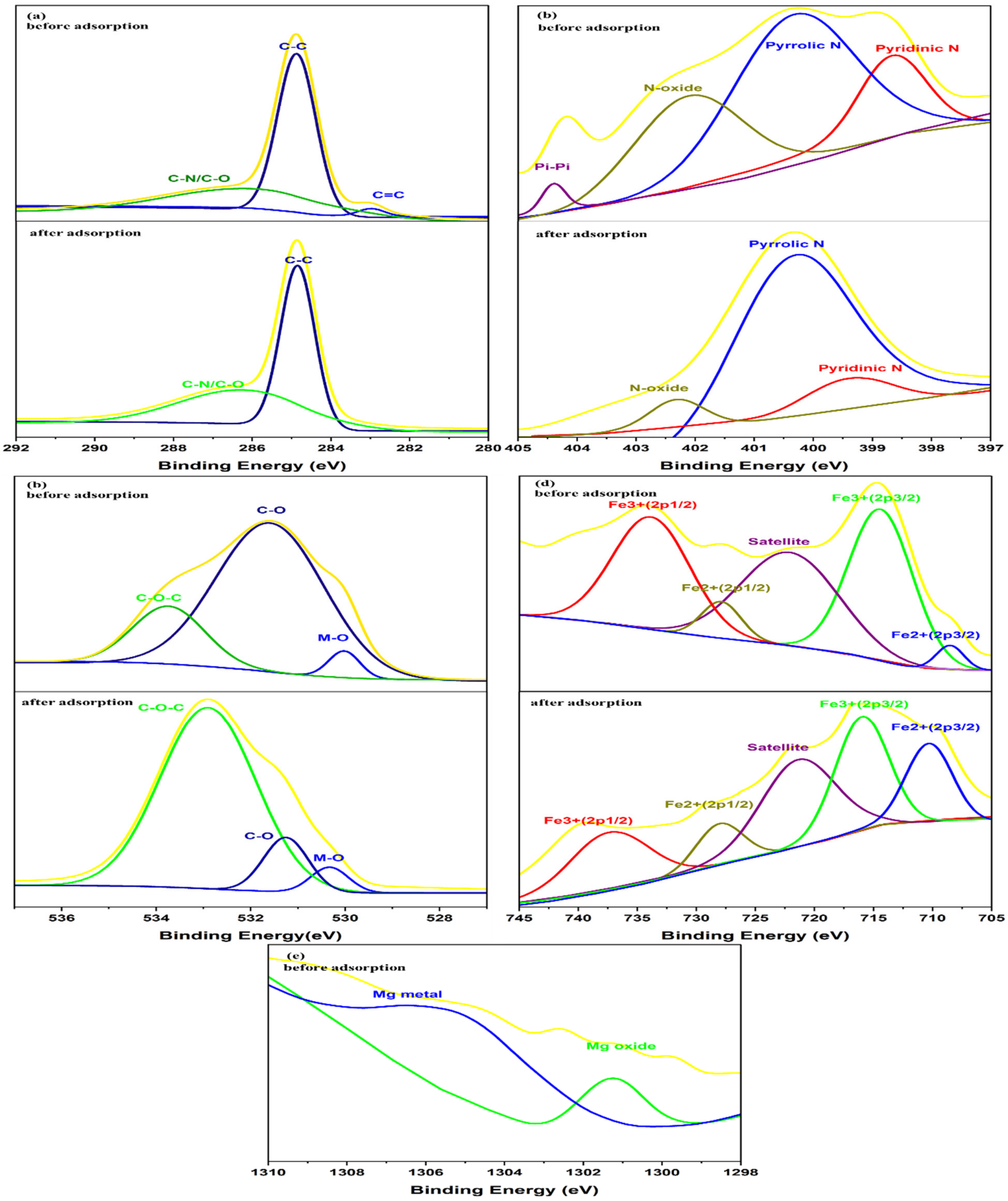

Fig. S2. High-resolution XPS spectra in (a) C 1s region, (b) O 1s region, (c) N 1s region, (d) Fe 2p region, (e) Mg 1s region. (The XPS spectra were plotted using OriginPro.)

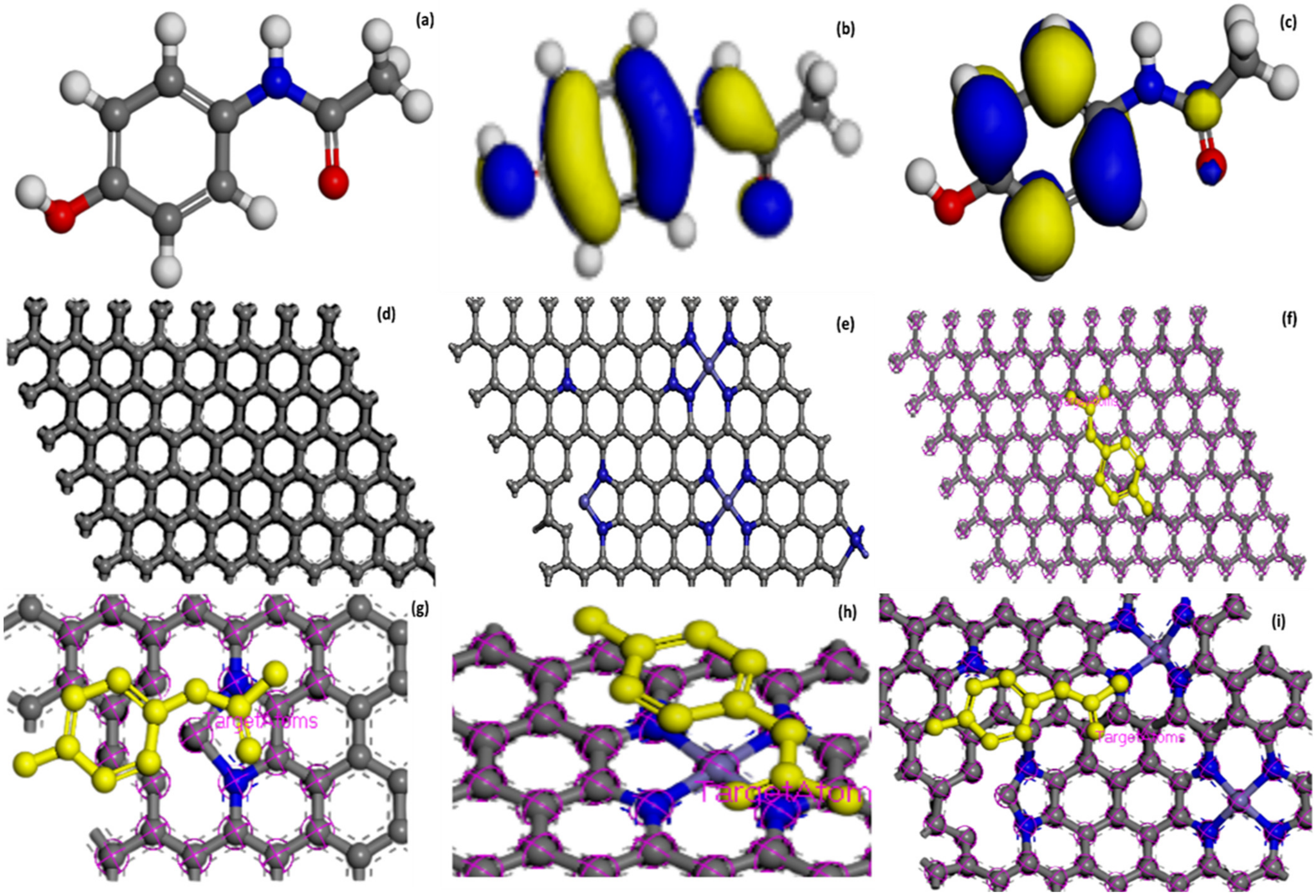

Fig. S3. Adsorption locator prediction of the most favorable adsorption sites of paracetamol on pristine biochar (a) Structure of PCM, (b) HOMO of PCM, (c) LUMO of PCM, (d) BC, (e) Fe-Mg@N-BC-3, (f) PCM&BC, (g) PCM&Mg-NBC, (h) PCM&Fe-NBC, and (i) PCM&Fe-Mg@N-BC-3.

Reference

(1) Wang, J.; Guo, X. Adsorption isotherm models: Classification, physical meaning, application and solving method. *Chemosphere* **2020**, *258*, 127279. DOI: 10.1016/j.chemosphere.2020.127279.

(2) Kalam, S.; Abu-Khamsin, S.A.; Kamal, M.S.; Patil, S. Surfactant Adsorption Isotherms: A Review. *ACS Omega* **2021,** *6,* 32342–32348. DOI:10.1021/acsomega.1c04661.

(3) VINCO, J.H.; BOTELHO JUNIOR, A.B.; DUARTE, H.A.; ESPINOSA, D.C.R.; TENÓRIO, J.A.S. Kinetic modeling of adsorption of vanadium and iron from acid solution through ion exchange resins. *Transactions of Nonferrous Metals Society of China* **2022**, *32*, 2438–2450. DOI:10.1016/S1003-6326(22)65916-8.

(4) Allaoui, I.; Elmourabit, M.; Arfoy, B.; Hadri, M.; Barhoun, A.; Draoui, K. Adsorption equilibrium, kinetic, and thermodynamic studies on the removal of paracetamol from wastewater using natural and HDTMA-modified clay. *Desalination Water Treat.* **2024***, 318*, 100345. DOI: 10.1016/j.dwt.2024.100345.

(5) Al-Harby, N.F.; Albahly, E.F.; Mohamed, N.A.; Kinetics, Isotherm and Thermodynamic Studies for Efficient Adsorption of Congo Red Dye from Aqueous Solution onto Novel

Cyanoguanidine-Modified Chitosan Adsorbent. *Polymers (Basel).* **2021**, *13*. DOI:10.3390/polym13244446.

(6) Liang, D.; Ji, B.; Wang, Y.; Li, X.; Gao, W.-Y. Effect of activated carbon microstructure and adsorption mechanism on the efficient removal of chlorophyll a and chlorophyll b from Andrographis paniculata extract. *Sci. Rep.* **2023**, *13*, 21930. DOI:10.1038/s41598-023-42011-6.

(7) Zhou, X.; Yu, X.; Maimaitiniyazi, R.; Zhang, X.; Qu, Q. Discussion on the thermodynamic calculation and adsorption spontaneity re Ofudje et al. (2023). *Heliyon* **2024**, *10,* e28188. DOI: 10.1016/j.heliyon. 2024.e28188.
